# Precise zonal diagnosis: multi-*b*-value DWI model reveals differential predictors of clinically significant prostate cancer in peripheral and transition zones

**DOI:** 10.1186/s13244-026-02324-2

**Published:** 2026-06-05

**Authors:** Kangwen He, Yinsong Chen, Zhen Kang, Shichao Li, Mengmeng Gao, Ting Yin, Wei Chen, Omar Darwish, Zhen Li

**Affiliations:** 1 Department of Radiology, Tongji Hospital, Tongji Medical College, Huazhong University of Science and Technology, Wuhan, China; 2 MR Research Collaborations, Siemens Healthineers Ltd., Chengdu, China; 3 MR Research Collaboration Team, Siemens Healthineers Ltd., Guangzhou, China; 4 MR Application Predevelopment, Siemens Healthineers Ltd., Erlangen, Germany

**Keywords:** Prostate cancer, Magnetic resonance imaging, Advanced diffusion models, Zone-specific diagnosis

## Abstract

**Objectives:**

To evaluate the diagnostic performance of multi-*b*-value DWI models for clinically significant prostate cancer (csPCa), identify zone-specific predictors in the peripheral (PZ) and transition zones (TZ), and validate the model’s robustness across different MRI vendors.

**Materials and methods:**

This retrospective study enrolled 238 patients, comprising a primary cohort (*n* = 162) and an independent cross-vendor validation cohort (*n* = 76). Seven diffusion models (mono-exponential model (MEM), intravoxel incoherent motion (IVIM), diffusion kurtosis imaging (DKI), stretched-exponential model (SEM), fractional order calculus (FROC), continuous-time random walk (CTRW), and IVIM-DKI model) were fitted to generate 18 parameters. LASSO regression and generalized estimating equations (GEE) identified independent predictors. Model performance was assessed using ROC curves and decision curve analysis (DCA). Subgroup analyses were performed in PZ/TZ.

**Results:**

MEM_ADC and CTRW_alpha were identified as robust independent predictors of csPCa. In the test set of the primary cohort, the Clinical+Multib_DWI model achieved an AUC of 0.85. Although the improvement over the Clinical+ADC model (AUC = 0.80) was not statistically significant (*p* > 0.05), the multi-*b*-value model demonstrated superior clinical net benefit. Crucially, in the cross-vendor validation cohort, the model maintained robust diagnostic accuracy (AUC = 0.88). Subgroup analysis revealed that CTRW_alpha exhibited strong diagnostic value for TZ lesions (AUC = 0.86) and TZ PI-RADS 3 lesions (AUC = 0.82).

**Conclusion:**

MEM_ADC and CTRW_alpha are zone-specific predictors of csPCa. While the multi-*b*-value model did not significantly outperform the ADC model in AUC, it offered superior clinical utility through higher net benefit and demonstrated cross-vendor robustness, supporting the translational potential of advanced diffusion models.

**Critical relevance statement:**

This study identifies zone-specific diffusion predictors for prostate cancer. By demonstrating robustness across different MRI vendors, the findings demonstrate that advanced diffusion models can be successfully translated from specialized protocols to clinical settings, providing superior decision-making utility regarding biopsy necessity.

**Key Points:**

- Advanced diffusion models lack cross-vendor validation for prostate cancer diagnosis.
- Selected diffusion parameters demonstrated robust cancer prediction across independent scanner vendors.
- Zone-specific evaluation offers superior clinical benefit for personalized biopsy decisions.

**Graphical Abstract:**

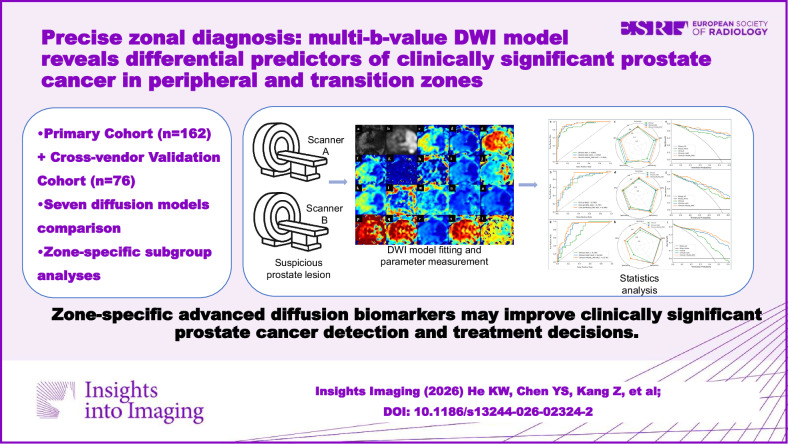

## Introduction

Prostate cancer (PCa) is a leading cause of cancer-related mortality in men [1]. The precise identification of clinically significant PCa (csPCa), defined by the International Society of Urological Pathology (ISUP) as Gleason grade (GG) ≥ 2, is critical to balance the risks of underdiagnosis of aggressive disease against overtreatment of indolent tumors [2]. Current diagnostic paradigms relying on prostate-specific antigen (PSA) testing and prostate biopsy remain constrained by limited specificity and sampling inaccuracies, contributing to both missed diagnoses and unnecessary interventions [3].

Multiparametric MRI (mpMRI) has transformed PCa evaluation, enabling non-invasive detection and risk stratification. Standard mpMRI protocol integrates T2-weighted imaging (T2WI), diffusion-weighted imaging (DWI) with derived apparent diffusion coefficient (ADC) maps, and dynamic contrast-enhanced sequences, as outlined in the Prostate Imaging-Reporting and Data System version 2.1 (PI-RADS v2.1) [4]. DWI/ADC serves as a cornerstone for csPCa identification, particularly in the peripheral zone (PZ), by capturing microstructural alterations in cellularity and tissue organization [5, 6]. Clinically, it is primarily interpreted in a qualitative manner. However, the conventional mono-exponential model (MEM) assumes Gaussian water diffusion—an oversimplification that fails to reflect the complex, heterogeneous microenvironment of PCa [6], limiting diagnostic accuracy especially in challenging scenarios like transition zone (TZ) lesions and equivocal PI-RADS 3 cases [7], even when quantitative analysis is employed.

Advanced multi-*b*-value DWI models address this limitation by quantifying non-Gaussian diffusion. While standard ADC can also be derived from these multi-*b*-value acquisitions as a comparative baseline, advanced techniques—including intravoxel incoherent motion (IVIM) model [8], diffusion kurtosis imaging (DKI) [9], stretched-exponential model (SEM) [10], fractional order calculus (FROC) [11], continuous-time random walk (CTRW) [12], and hybrid IVIM-DKI [13]—extract further microstructural information and have shown promise in tumor characterization [14–16] and other pathologies [17, 18]. Yet their comparative utility in PCa remains inadequately explored, with prior studies constrained by small cohorts, isolated model evaluation, or insufficient subgroup analyses [19–21].

To bridge this gap, we systematically compared seven multi-*b*-value DWI models in a substantial cohort. Our step-wise analytical pipeline encompassed: (1) overall and zone-specific evaluation of diffusion parameters for csPCa detection; (2) selection of robust imaging predictors; and (3) construction and evaluation of multiparametric models integrating optimal diffusion features with clinical variables. By defining a precision imaging framework tailored to the anatomical context, this work seeks to advance personalized PCa diagnosis.

## Methods

### Patients

This retrospective study was approved by the institutional review board (TJ-IRB202404076), and the written informed consent was waived. For the primary cohort, between December 2023 and January 2025, 245 patients with suspected PCa underwent 3.0 T mpMRI, including a 12 *b*-value DWI sequence, followed by prostate biopsy within two weeks. The exclusion criteria were: (1) prior intervention before MRI (*n* = 25), (2) clinically diagnosed acute prostatitis (*n* = 15), as prostate biopsy is contraindicated during the active infection phase due to the risk of sepsis [22], and acute inflammation can induce transient PSA elevation and diffuse MRI artifacts that confound cancer detection, (3) incomplete imaging protocols (*n* = 33), and (4) severe image artifacts, including significant geometric distortions on high *b*-value DWIs (*n* = 10). Ultimately, 162 patients with 224 MRI-suspicious lesions were included as the primary dataset. Patients were randomly allocated at the patient level into the training set (*n* = 113; 70%) and the test set (*n *= 49; 30%).

To evaluate model generalizability, an independent cross-vendor validation cohort (initial *n* = 201) was retrospectively collected between August 2015 and January 2018 using another MR scanner. Applying identical inclusion and exclusion criteria, 76 patients with 84 MRI-suspicious lesions were included in this validation set.

**Fig. 1:**
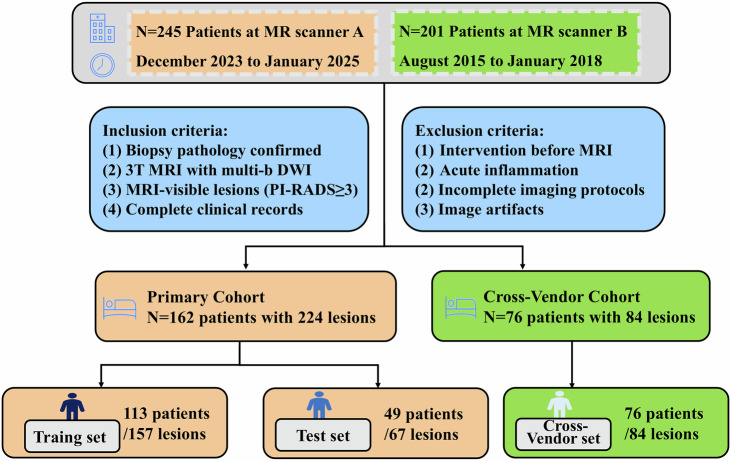
Flowchart of the patient enrollment

The patient selection flowchart for both cohorts is shown in Fig. 1.

### Pathology

For the primary cohort, all patients underwent transperineal MRI-ultrasound fusion-guided targeted plus 12-core systematic biopsy within 2 weeks after MRI. In contrast, the cross-vendor validation cohort relied on a standard 12-core systematic biopsy protocol. To ensure label accuracy for this cohort, radiological-pathological correlation was established based on strict spatial concordance between the MRI index lesion location and the corresponding positive or negative systematic biopsy sector. Each specimen was labeled by anatomical location. To maintain a strictly lesion-based focus, MRI-invisible tumors (positive systematic biopsy cores lacking corresponding mpMRI-suspicious lesions) were excluded from quantitative analysis. Pathological assessment was performed using the Gleason grading system defined by ISUP [2]. CsPCa was defined as an ISUP Gleason score ≥ 3 + 4, whereas a Gleason score of 3 + 3 or benign findings were classified as non-csPCa.

### MRI acquisition

MRI examinations were performed on 3.0 T scanners: MAGNETOM Skyra (Siemens Healthineers) using a reduced-field-of-view DWI sequence (ZOOMit) for the primary cohort, and Discovery MR750 (GE Healthcare) using a standard multi-*b*-value DWI protocol for the cross-vendor validation cohort. Both protocols included high *b*-values up to 3000 s/mm². Detailed acquisition parameters for both scanners are listed in Supplementary Methods.

### Image analysis

Seven diffusion models were fitted: MEM, SEM, IVIM, DKI, IVIM-DKI, CTRW, and FROC. The IVIM-DKI model was implemented with a publicly available MATLAB toolbox [23]. The remaining six models were fitted using the Body-DiffusionLab module of the MR Station platform (Chengdu ZhongYing Medical Technology Co., Ltd.). Parameters were denoted as ModelName_ParameterName, yielding 18 quantitative metrics. Detailed mathematical formulations, *b*-value selections, and the complete parameter list are provided in the Supplementary Materials.

**Fig. 2:**
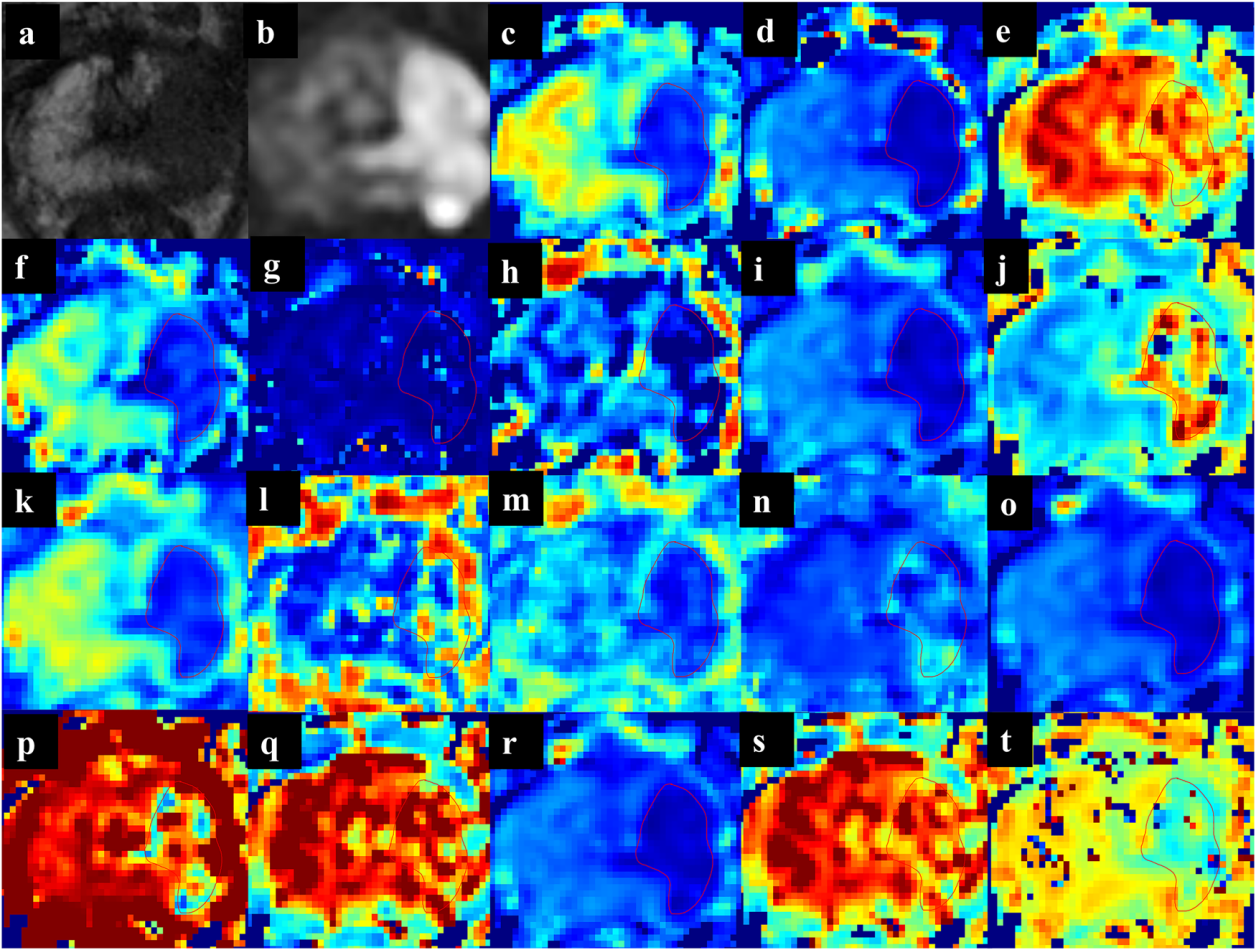
Parameter maps of different DWI models. A 73-year-old man with a serum PSA level of 30.932 ng/mL showing a PI-RADS category 5 lesion located in the left apex of the peripheral zone. Representative images of the lesion at its largest cross-section are shown. **a** Axial T2WI; **b** DWI at *b* = 1500 s/mm²; (**c**–**t**) Corresponding parametric maps derived from different DWI models, including MEM_ADC, SEM_DDC, SEM_alpha, IVIM_D, IVIM_Dstar, IVIM_f, DKI_D, DKI_K, IVIM-DKI_D, IVIM-DKI_Dp, IVIM-DKI_f, IVIM-DKI_K, CTRW_D, CTRW_alpha, CTRW_beta, FROC_D, FROC_beta, FROC_mu

Two radiologists (5 and 9 years of prostate MRI interpretation experience) performed the ROI delineation independently. To mimic the clinical scenario while maintaining blinding to outcomes, a research coordinator provided the readers with the anatomical location of the targeted lesions or positive systematic biopsy sectors derived from the original biopsy procedure logs. Guided by the localization information, readers delineated volumetric whole-lesion ROIs based on biparametric MRI, continuously cross-referencing T2WI and DWI to accurately define anatomical boundaries. These ROIs, drawn within the MEM_ADC space using ITK-SNAP (v3.8), were visually checked to exclude any peripheral regions affected by high *b*-value geometric distortions before being co-registered to all other maps (Fig. 2). Crucially, both readers were blinded to the final histopathological results during the measurement process. To reduce partial-volume effects, only lesions with a short-axis diameter ≥ 3 mm were included. Inter-observer intraclass correlation coefficients (ICCs) were calculated independently for all 18 parameters due to their varying sensitivities to ROI boundary shifts. To ensure feature robustness against the inherent noise of non-Gaussian models, we applied a strict reliability threshold of ICC ≥ 0.80 (exceeding the conventional 0.75) [24, 25]. The average of the two readers’ measurements was used for analysis. Morphological characteristics, including maximum diameter and lesion volume derived from the ROI segmentation, were similarly averaged between the two readers.

### Statistics

In the primary cohort, demographic and imaging characteristics were compared using Mann–Whitney *U* or chi-square tests, with false discovery rate (FDR) adjustment for multiple comparisons between csPCa and non-csPCa. Subgroup analyses were performed by zones (TZ or PZ) and PI-RADS categories. An additional analysis focused on PI-RADS 3 lesions in TZ, which are considered equivocal in prostate MRI interpretation [26]. Spearman’s rank correlation coefficients were calculated to evaluate the association between diffusion parameters and GG in PZ and TZ.

Least absolute shrinkage and selection operator (LASSO) regression with five-fold cross-validation was applied in the training set to select robust imaging predictors from the 18 quantitative diffusion parameters. To ensure parameter stability, 500 bootstrap resamples were performed, and parameters retained in more than 50% of bootstrap iterations were considered stable. Generalized estimating equations (GEE) were then used to evaluate the independent association of the selected diffusion parameters with csPCa after adjusting for clinical covariates. GEE is particularly suitable for clustered or repeated-measures data, as it provides population-averaged estimates without strict distributional assumptions [27], making it ideal for adjusting intra-subject correlations arising from multiple lesions within the same patient.

To quantify the diagnostic value of the advanced diffusion metrics, three multivariable logistic regression models were constructed: (1) the Clinical model included age, prostate-specific antigen density (PSAD), digital rectal examination (DRE) status, anatomical zone, lesion volume, and PI-RADS score; (2) the Clinical+ADC model consisted of all the clinical variables plus the MEM_ADC; and (3) the Clinical+Multib_DWI model comprised the clinical variables integrated with the optimal stable diffusion predictors selected by the LASSO pipeline. Additionally, prompted by the spatial heterogeneity of diffusion parameters, an exploratory zone-specific split model (Unified Zone-Aware Model) was constructed to evaluate the theoretical potential of location-dependent parameter selection. Detailed methodology for this exploratory analysis is provided in the Supplementary Materials. ROC analysis was performed to evaluate the diagnostic performance of each model by calculating AUC, sensitivity, specificity, accuracy, positive predictive value (PPV), and negative predictive value (NPV). Model comparisons were conducted using the DeLong test. Clinical utility was further evaluated with decision curve analysis (DCA).

For the cross-vendor validation cohort, ComBat harmonization [28] was performed to minimize non-biological scanner effects. Parameter consistency between the primary (Siemens) and validation (GE) cohorts within the same pathological groups was checked using the Mann-Whitney U test. The training-derived models were then applied to the validation cohort to assess diagnostic performance and clinical utility.

## Results

### Participant characteristics

A total of 238 eligible patients were enrolled in this study and categorized into a primary cohort (*n* = 162 patients with 224 lesions) for parameter comparison and model development, and an independent cross-vendor validation cohort (*n* = 76 patients with 84 lesions) for robustness testing. In the 224 MRI-suspicious lesions of the primary cohort (median age, 67 years; IQR, 61.3-73.0), 99 (44.2%) were located in PZ and 125 (55.8%) in TZ. No significant differences in baseline characteristics were observed between training and test sets (Table 1). The detailed clinical and pathological characteristics of the primary and validation cohorts are summarized in Table 1.

**Table 1:** Characteristics of patients and lesions enrolled in this study

Patient level	Primary cohort		Validation cohort	
Variables	training set (*n* = 113)	test set (n = 49)	cross-vendor set (*n* = 76)	p^*^
Age (year)	67.00 (62.00–73.00)	67.00 (60.00–73.00)	65.50 (61.75–73.00)	0.445
PSA (ng/mL)	13.27 (8.34–23.21)	12.70 (8.75–26.29)	13.72 (8.28–41.88)	0.8659
DRE				0.608
0	58 (51.3)	23 (46.9)	32 (42.1)	
1	55 (48.7)	26 (53.1)	44 (57.9)	
Volume (mL)	45.61 (30.79–63.15)	44.64 (29.03–54.92)	48.31 (31.46–73.59)	0.229
PSAD (ng/mL^2^)	0.29 (0.20–0.67)	0.42 (0.17–0.94)	0.35 (0.14–0.91)	0.354
Lesion number				0.624
Single	81(71.7)	34 (69.4)	68 (89.5)	
Multiple	32 (28.3)	15 (30.6)	8 (10.5)	
Lesion level				
**Variables**	**training set (*n*** = **157)**	**test set (*n*** = **67)**	**cross-vendor set (*n*** = **84)**	p^*^
Lesion diameter (cm)	1.98 (1.46–2.78)	1.70 (1.36–2.64)	1.97 (1.47–3.09)	0.237
Lesion volume (cm^3^)	1.65 (0.79–4.19)	1.31 (0.67–3.93)	1.83 (0.64–5.52)	0.327
Zone				0.928
PZ	69 (43.9)	30 (44.8)	42 (50.0)	
TZ	88 (56.1)	37 (55.2)	42 (50.0)	
PI-RADS				0.255
3	86 (54.8)	32 (47.8)	43 (51.2)	
4	29 (18.5)	19 (28.3)	21 (25.0)	
5	42 (26.7)	16 (23.9)	20 (23.8)	
GG				0.238
0	67 (42.7)	21 (31.3)	41 (48.8)	
1	17 (10.8)	12 (17.9)	6 (7.1)	
2	23 (14.6)	14 (20.9)	7 (8.3)	
3	20 (12.7)	12 (17.9)	7 (8.3)	
4	12 (7.6)	3 (4.5)	14 (16.7)	
5	18 (11.5)	5 (7.5)	9 (10.7)	
csPCa				0.860
0	84 (53.5)	33 (49.3)	47 (56.0)	
1	73 (46.5)	34 (50.7)	37 (44.0)	

Continuous variables are expressed as median (interquartile range), while categorical variables are expressed as frequency (percentage). The Mann–Whitney *U*-test and chi-square test were used for hypothesis testing of continuous and categorical variables, respectively

p* represents the *p*-value after correction for multiple comparisons using the False Discovery Rate (FDR) method, and indicates statistical comparisons between the training and test sets to verify the randomness of the dataset split

*PSA* prostate-specific antigen, *PSAD* prostate-specific antigen density, *DRE* digital rectal examination, *PZ* peripheral zone, *TZ* transitional zone, *PI-RADS* Prostate Imaging-Reporting and Data System, *csPCa* clinically significant prostate cancer, *GG* Gleason grade

### Comparisons of diffusion parameters

**Fig. 3:**
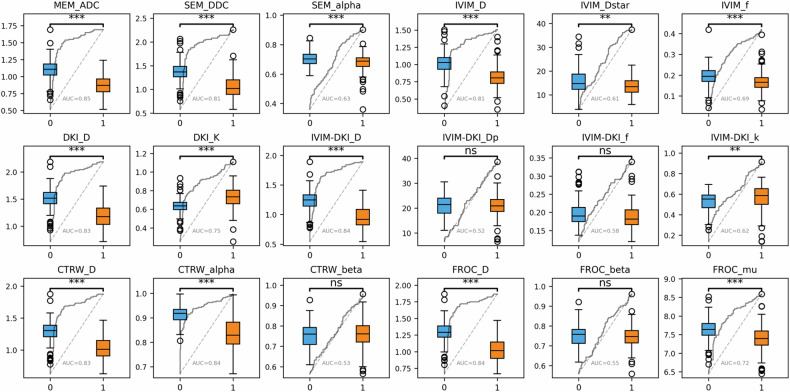
Boxplots of DWI parameters compared in csPCa (1) and non-csPCa (0) with embedded ROC curves and annotated AUC values. Units: MEM_ADC, SEM_DDC, IVIM_D, IVIM_Dstar, DKI_D, IVIM_DKI_D, IVIM_DKI_Dp, CTRW_D, and FROC_D are expressed in ×10⁻³ mm²/s; FROC_μ in μm; SEM_Alpha, IVIM_f, DKI_K, IVIM-DKI_f, IVIM-DKI_k, FROC_β, CTRW_α, and CTRW_β are dimensionless. AUC, area under the curve. “ns”, “*”, “**”, and “***” indicate FDR-adjusted *p*-values ≥ 0.05, < 0.05, < 0.01, and < 0.001, respectively

All diffusion parameters demonstrated excellent interobserver reproducibility (ICCs > 0.80, Table S1). Among all 224 lesions, all parameters—except IVIM-DKI_Dp, IVIM-DKI_f, CTRW_beta, and FROC_beta—showed significant differences between csPCa and non-csPCa after FDR correction (*p* < 0.05) (Table 2). MEM_ADC, IVIM-DKI_D, CTRW_alpha, and FROC_D demonstrated the highest diagnostic performance, with AUCs of 0.85, 0.84, 0.84, and 0.84, respectively (Fig. 3).

**Table 2:** Comparison of the radiographic characteristics and DWI model parameters for distinguishing csPCa lesions in the primary cohort (overall and stratified by lesion location)

MRI Parameters	Overall			PZ			TZ			PZ vs. TZ	
	csPCa (*n* = 107)	Non-csPCa (*n* = 117)	*P*^*a*^	csPCa (*n* = 61)	Non-csPCa (*n* = 38)	*P*^*a*^	csPCa (*n* = 46)	Non-csPCa (*n* = 79)	*P*^*a*^	*P*^*b*^	*P*^*c*^
Lesion diameter (cm)	2.38 (1.57–3.28)	1.61 (1.34–2.12)	< 0.001^*^	2.13 (1.51–3.04)	1.54 (1.31–2.05)	0.019^*^	2.76 (2.07–4.09)	1.65 (1.34–2.16)	< 0.001^*^	0.133	0.690
Lesion Volume (cm^3^)	2.94 (0.93–6.46)	1.10 (0.68–2.04)	< 0.001^*^	2.10 (0.70–4.76)	0.77 (0.53–1.91)	0.026^*^	4.32 (2.11–14.27)	1.23 (0.78–2.18)	< 0.001^*^	0.027	0.144
PI-RADS			< 0.001^*^			< 0.001^*^			< 0.001^*^	0.770	0.455
3	24 (22.4)	100 (85.5)		17 (27.9)	31 (81.6)		7 (15.2)	69 (87.3)			
4	36 (33.6)	12 (10.3)		20 (32.8)	6 (15.8)		16 (34.8)	6 (7.6)			
5	47 (43.9)	5 (4.3)		24 (39.3)	1 (2.6)		23 (50.0)	4 (5.1)			
MEM_ADC	0.87 (0.78–0.97)	1.11 (1.02–1.19)	< 0.001^*^	0.88 (0.76–0.96)	1.11 (1.00-1.22)	< 0.001^*^	0.84 (0.80–0.97)	1.11 (1.04–1.18)	< 0.001^*^	0.851	0.907
SEM_DDC	1.02 (0.89–1.20)	1.37 (1.26–1.48)	< 0.001^*^	1.07 (0.89–1.27)	1.42 (1.22-1.54)	< 0.001^*^	0.99 (0.90–1.12)	1.36 (1.29–1.47)	< 0.001^*^	0.774	0.642
SEM_alpha	0.69 (0.65–0.71)	0.70 (0.67–0.73)	0.0011^*^	0.68 (0.64–0.70)	0.71 (0.67–0.74)	0.010^*^	0.69 (0.66–0.71)	0.70 (0.67–0.73)	0.108	0.769	0.907
IVIM_D	0.81 (0.72–0.89)	1.03 (0.93–1.10)	< 0.001^*^	0.80 (0.71–0.91)	1.04 (0.93–1.14)	< 0.001^*^	0.81 (0.74–0.89)	1.03 (0.94–1.09)	< 0.001^*^	0.851	0.690
IVIM_Dstar	13.43 (11.07–15.90)	14.73 (12.31–18.72)	0.005^*^	13.41 (11.07–15.95)	14.40 (11.89–18.45)	0.383	13.52 (11.19–15.75)	15.10 (12.48–18.93)	0.013^*^	0.910	0.690
IVIM_f	0.16 (0.14–0.19)	0.19 (0.17–0.22)	< 0.001^*^	0.17 (0.14–0.20)	0.18 (0.15–0.21)	0.182	0.16 (0.14–0.18)	0.20 (0.17–0.23)	< 0.001^*^	0.769	0.281
DKI_D	1.18 (1.03–1.33)	1.52 (1.42–1.62)	< 0.001^*^	1.20 (1.01–1.37)	1.53 (1.34–1.62)	< 0.001^*^	1.14 (1.07–1.24)	1.51 (1.44–1.62)	< 0.001^*^	0.851	0.907
DKI_K	0.73 (0.66–0.80)	0.64 (0.60-0.68)	< 0.001^*^	0.72 (0.65–0.80)	0.62 (0.55–0.66)	< 0.001^*^	0.74 (0.68–0.81)	0.64 (0.61–0.69)	< 0.001^*^	0.769	0.144
IVIM-DKI_D	0.92 (0.83–1.09)	1.25 (1.14–1.33)	< 0.001^*^	0.93 (0.82–1.10)	1.25 (1.10–1.33)	< 0.001^*^	0.91 (0.85–1.07)	1.24 (1.14-1.32)	< 0.001^*^	0.858	0.907
IVIM-DKI_Dp	20.93 (18.68–23.46)	21.46 (17.95–24.02)	0.615	20.56 (18.51–23.15)	19.61 (16.10–22.24)	0.271	21.65 (19.20–24.14)	21.85 (19.57–24.37)	0.578	0.769	0.144
IVIM-DKI_f	0.18 (0.17–0.21)	0.19 (0.17–0.21)	0.056	0.19 (0.17–0.22)	0.21 (0.18–0.22)	0.151	0.17 (0.16–0.20)	0.18 (0.17–0.21)	0.017^*^	0.179	0.144
IVIM-DKI_K	0.59 (0.50–0.65)	0.55 (0.47–0.59)	0.003^*^	0.56 (0.50–0.65)	0.50 (0.44–0.57)	0.004^*^	0.60 (0.53–0.67)	0.56 (0.51–0.60)	0.022^*^	0.769	0.144
CTRW_D	1.01 (0.91–1.15)	1.30 (1.21–1.39)	< 0.001^*^	1.02 (0.90–1.18)	1.31 (1.15–1.42)	< 0.001^*^	0.98 (0.92–1.14)	1.30 (1.23–1.38)	< 0.001^*^	0.851	0.907
CTRW_alpha	0.83 (0.79–0.88)	0.92 (0.89–0.93)	< 0.001^*^	0.84 (0.79–0.89)	0.90 (0.88–0.93)	< 0.001^*^	0.82 (0.79–0.88)	0.92 (0.90–0.94)	< 0.001^*^	0.851	0.144
CTRW_beta	0.76 (0.72–0.80)	0.76 (0.71–0.79)	0.480	0.76 (0.72–0.80)	0.77 (0.73–0.81)	0.290	0.77 (0.73–0.80)	0.75 (0.71–0.79)	0.155	0.851	0.144
FROC_D	1.01 (0.90–1.14)	1.29 (1.22–1.39)	< 0.001^*^	1.03 (0.88–1.18)	1.30 (1.15–1.40)	< 0.001^*^	1.01 (0.92–1.07)	1.29 (1.23–1.37)	< 0.001^*^	0.851	0.907
FROC_beta	0.75 (0.71–0.78)	0.76 (0.71–0.78)	0.225	0.75 (0.71–0.78)	0.77 (0.75–0.80)	0.034^*^	0.74 (0.72–0.76)	0.74 (0.71–0.78)	0.813	0.910	0.144
FROC_mu	7.40 (7.22–7.59)	7.64 (7.48–7.80)	< 0.001^*^	7.40 (7.22–7.62)	7.59 (7.45–7.78)	0.002^*^	7.39 (7.24–7.56)	7.66 (7.49–7.80)	< 0.001^*^	0.971	0.791

*P*^*a*^, the comparison of csPCa and non-csPCa between the global, PZ, or TZ group

*P*^*b*^, the comparison of csPCa between the PZ and TZ groups

*P*^*c*^, the comparison of non-csPCa between the PZ and TZ groups

*p*-values with an asterisk (*) superscript indicate statistical significance

All diffusion-related parameters are presented as median (interquartile range)

Statistical comparisons were performed using the Mann–Whitney *U*-test, and the False Discovery Rate (FDR) method was used for multiple comparisons

Units: MEM_ADC, IVIM_D, IVIM_Dstar, DKI_D, SEM_DDC, FROC_D, CTRW_D: ×10⁻³ mm²/s; FROC_mu: μm⁻¹; DKI_K, SEM_Alpha, FROC_beta, CTRW_alpha, CTRW_beta, IVIM_f: unitless

### Subgroup analysis by lesion location and PI-RADS category

In PZ, MEM_ADC achieved the highest AUC (0.84), followed by IVIM-DKI_D (AUC = 0.82) and CTRW_D, FROC_D and IVIM_D (all AUC = 0.81). In TZ, however, FROC_D and IVIM-DKI_D yielded the highest AUCs (0.87), followed by DKI_D, CTRW_D and CTRW_alpha (all AUC = 0.86). For PI-RADS 3 lesions, MEM_ADC best discriminated csPCa (AUC = 0.79), whereas CTRW_alpha was optimal for PI-RADS 4-5 lesions (AUC = 0.82) (Table 2, Table S2, and Figs. S1-S4).

### Correlation with GG

**Fig. 4:**
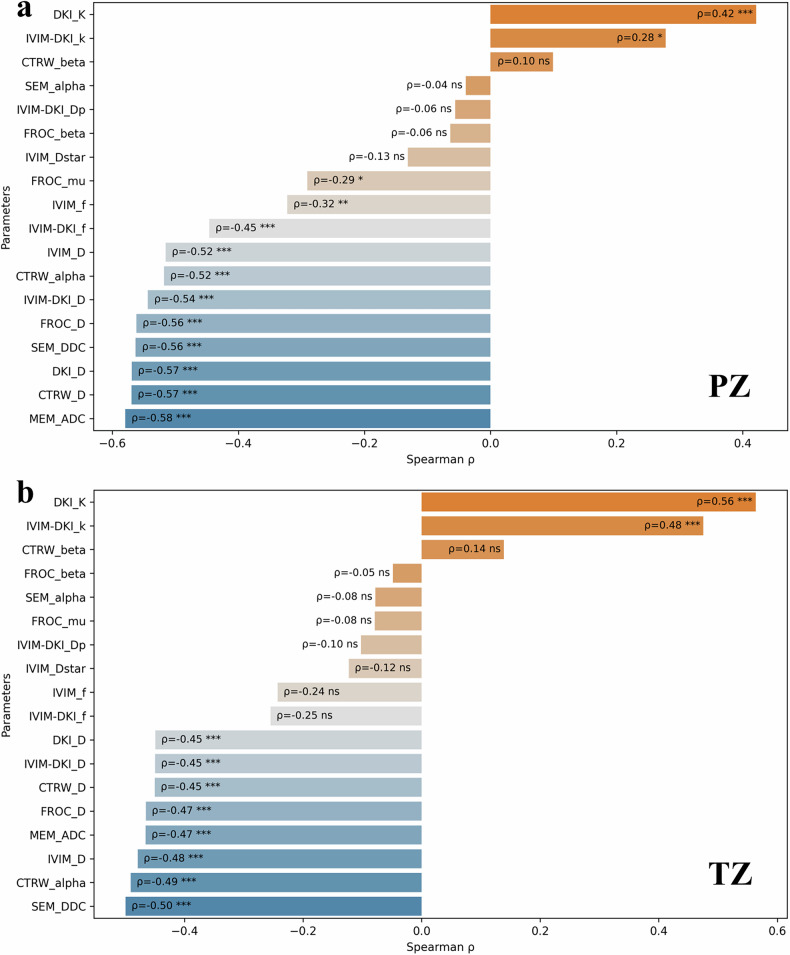
Spearman correlation analysis between DWI parameters and Gleason grade. (**a**) Peripheral zone (PZ). (**b**) Transition zone (TZ). The marks on each bar indicate the Spearman correlation coefficient (*ρ*) and statistical significance between each parameter and Gleason grade (GG). “ns”, “*”, “**”, and “***” indicate FDR-adjusted *p*-values ≥ 0.05, <  0.05, < 0.01, and < 0.001, respectively

In PZ, MEM_ADC demonstrated the strongest correlation with GG (ρ = −0.58, *p* < 0.001) (Fig. 4). In TZ, DKI_K correlated most strongly with GG (ρ = 0.56, *p* < 0.001). Across both zones, nine additional parameters (including CTRW_alpha) exhibited moderate correlations with GG. The absolute correlation coefficients for all these nine parameters ranged from 0.40 to 0.50 (all *p* < 0.001) (Fig. 4).

### Stable parameter selection

**Fig. 5:**
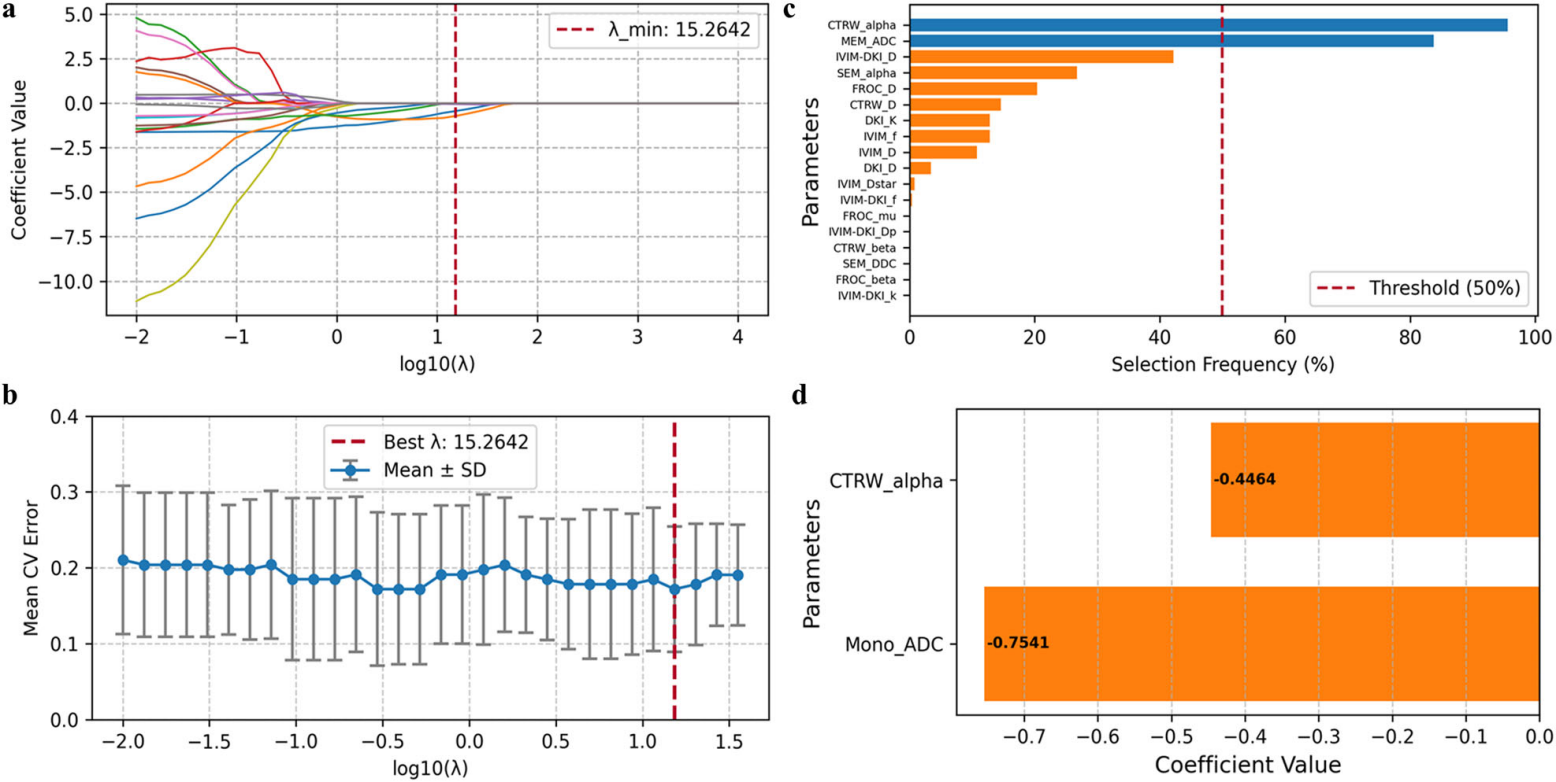
Logistic regression with LASSO and 500 bootstrap iterations to identify stable parameters. **a** Coefficient path plot: Shows how the coefficients of each variable shrink toward zero as the regularization strength (*λ*) increases, illustrating the variable selection process of LASSO. **b** Cross-validation curve: Five-fold cross-validation was performed, and the *λ* value corresponding to the lowest mean cross-validation error was selected as the optimal regularization parameter. **c** Bootstrap selection frequency: Parameters selected in more than 50% of the 500 bootstrap samples were considered stable and retained for the final model. **d** Final model coefficients: Displays the distribution of coefficients for the selected features in the final logistic regression model with the best lambda

LASSO regression with bootstrap identified CTRW_alpha and MEM_ADC as the most stable parameters, appearing in 95.6% and 83.8% of iterations, respectively (Fig. 5). GEE analysis showed that, after adjusting for clinical variables, both CTRW_alpha and MEM_ADC remained independent predictors of csPCa, with odds ratios of 0.365 (*p* = 0.007) and 0.358 (*p* = 0.038), respectively (Table 3).

**Table 3:** Generalized estimating equations analysis of clinical and diffusion parameters for predicting csPCa

Variables	Coefficient	OR	Std_error	*p*-value
Age	0.083	1.086	0.033	0.012^*^
PSAD	2.166	8.723	1.125	0.054
DRE	−0.947	0.388	0.619	0.126
PIRADS_4	1.777	5.909	0.846	0.036^*^
PIRADS_5	0.696	2.006	0.691	0.314
Zone	0.682	1.978	0.550	0.215
Lesion_volume	0.041	1.042	0.034	0.226
CTRW_alpha	−1.007	0.365	0.372	0.007^*^
MEM_ADC	−1.027	0.358	0.496	0.038^*^
intercept	−6.825	0.001	2.510	0.007^*^

PI-RADS scores were included as dummy variables in the model, with PI-RADS 3 as the reference group; PI-RADS_4 and PI-RADS_5 represent comparisons of scores 4 and 5 versus 3, respectively

*p*-values with an asterisk (*) superscript indicate statistical significance in the generalized estimating equations (GEE) analysis

### Model construction and evaluation

**Fig. 6:**
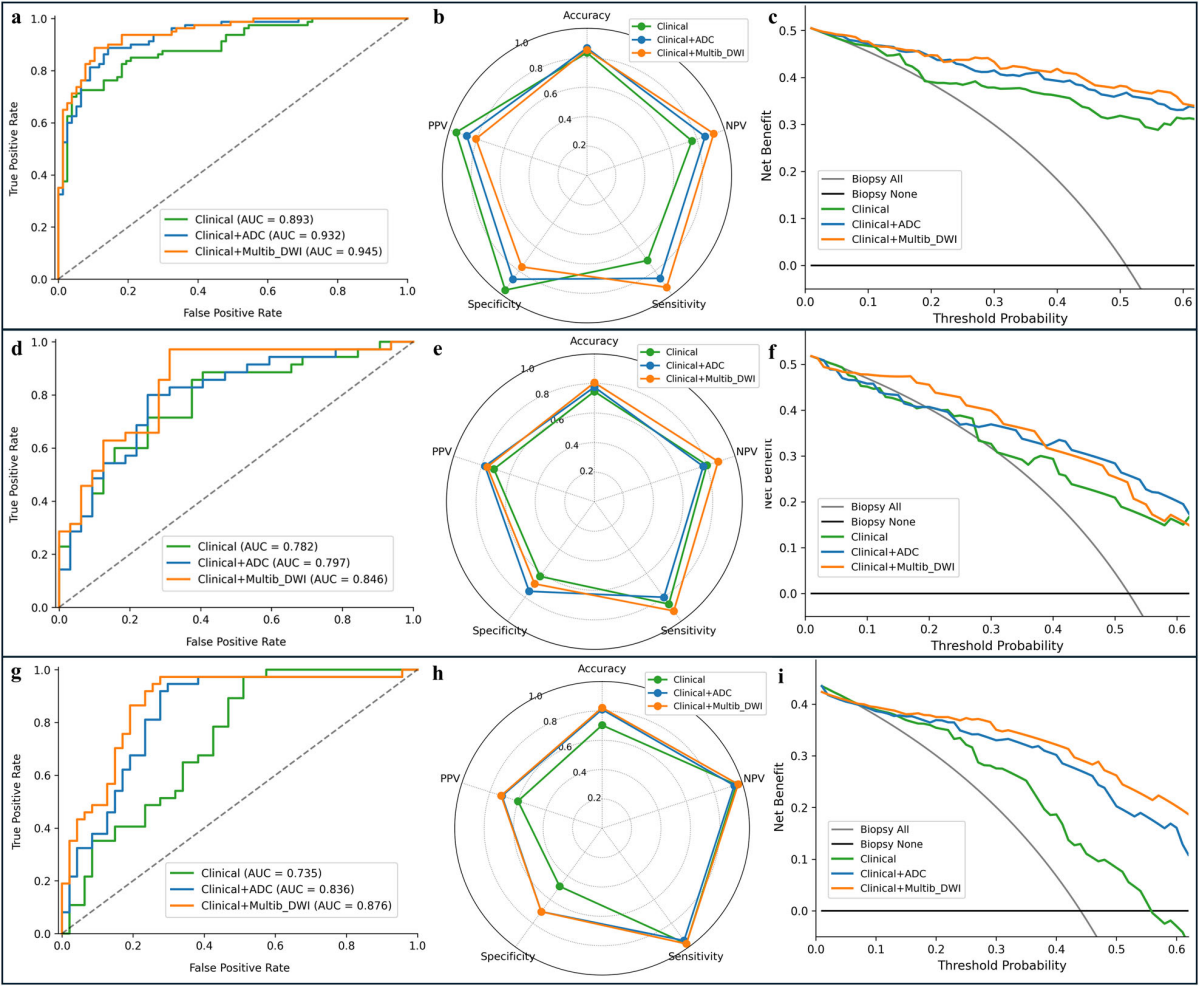
Diagnostic performance and clinical utility for distinguishing clinically significant prostate cancer (csPCa). Receiver operating characteristic curves, radar plots, and decision curve analysis of the clinical, clinical+ADC and clinical+Multib_DWI models for distinguishing between csPCa and non-csPCa in the training set (**a**–**c**), test set (**d**–**f**), and cross-vendor set (**g**–**i**)

In the test set, the Clinical+multib_DWI model achieved a higher AUC than the Clinical+ADC model (0.846 vs. 0.797, *p* > 0.05) (Table 4). Notably, the combined model demonstrated superior NPV (0.88 vs. 0.77) and sensitivity (0.91 vs. 0.80), with comparable PPV and specificity (Fig. 6). DCA indicated that in the test set, the Clinical+Multib_DWI model yielded a significantly higher net benefit than the other two models within the threshold probability range of 0.10 to 0.40 (Fig. 6).

**Table 4:** Comparison of models in the training, test, and cross-vendor set

Dataset	Model	AUC (95% CI)	Accuracy	Sensitivity	Specificity	PPV	NPV
Training set	Clinical	0.893 (0.836, 0.938)	0.83	0.70	0.96	0.95	0.76
	Clinical+ADC	0.932 (0.886, 0.966)	0.87	0.89	0.86	0.87	0.88
	Clinical+Multib_DWI	0.945 (0.905, 0.973)	0.85	0.94	0.75	0.80	0.92
Test set	Clinical	0.782 (0.668, 0.882)	0.75	0.86	0.63	0.71	0.80
	Clinical+ADC	0.797 (0.685, 0.892)	0.78	0.80	0.75	0.78	0.77
	Clinical+Multib_DWI	0.846 (0.749, 0.931)	0.81	0.91	0.69	0.76	0.88
Cross-vendor set	Clinical	0.735 (0.626, 0.832)	0.70	0.97	0.49	0.60	0.96
	Clinical+ADC	0.836 (0.738, 0.917)	0.81	0.95	0.70	0.71	0.94
	Clinical+Multib_DWI	0.876 (0.790, 0.947)	0.82	0.97	0.70	0.72	0.97

*AUC* area under the receiver operating characteristic curve, *PPV* positive predictive value, *NPV* negative predictive value

### Value of diffusion models in equivocal TZ PI-RADS 3 lesions

In TZ PI-RADS 3 lesions, IVIM_f best distinguished PCa from benign lesions (AUC = 0.79), whereas CTRW_alpha and FROC_D best discriminated csPCa from non-csPCa (AUC = 0.82 each) (Table 5; Figs.  S5 and S6). Multivariable analysis identified CTRW_alpha as a robust independent predictor for both PCa (*p* = 0.008) and csPCa (*p* = 0.004), while MEM_ADC associated only with PCa (*p* = 0.035) (Table S3).

**Table 5:** Comparison of the radiographic characteristics and DWI model parameters for distinguishing PCa and csPCa in PI-RADS category 3 TZ lesions within the primary cohort

MRI parameters	PCa (*n* = 17)	Non-PCa (*n* = 59)	*p*	csPCa (*n* = 9)	Non-csPCa (*n* = 67)	*p*
Lesion diameter	1.36 (1.12–1.98)	1.66 (1.34–2.14)	0.250	1.32 (0.93–2.19)	1.65 (1.33–1.99)	0.438
Lesion volume	0.76 (0.38–1.94)	1.31 (0.79–2.11)	0.149	0.76 (0.38–2.52)	1.22 (0.77–1.97)	0.438
MEM_ADC	1.03 (0.90–1.15)	1.12 (1.06–1.18)	0.045^*^	0.99 (0.88–1.11)	1.12 (1.05–1.18)	0.044^*^
SEM_DDC	1.29 (1.13–1.39)	1.36 (1.30–1.49)	0.045^*^	1.13 (1.12–1.36)	1.36 (1.30–1.47)	0.053
SEM_alpha	0.70 (0.66–0.73)	0.71 (0.68–0.73)	0.752	0.69 (0.65–0.72)	0.71 (0.68–0.73)	0.451
IVIM_D	1.03 (0.89–1.06)	1.03 (0.95–1.09)	0.701	1.03 (0.81–1.06)	1.03 (0.96–1.09)	0.465
IVIM_Dstar	14.57 (10.86–18.33)	15.24 (12.57–18.02)	0.721	11.56 (9.73–18.22)	15.68 (12.56–18.79)	0.343
IVIM_f	0.17 (0.16–0.18)	0.21 (0.18–0.23)	0.004^*^	0.17 (0.12–0.18)	0.20 (0.18–0.23)	0.044^*^
DKI_D	1.34 (1.20–1.52)	1.53 (1.46–1.65)	0.021^*^	1.26 (1.14–1.34)	1.53 (1.45–1.64)	0.028^*^
DKI_K	0.63 (0.58–0.68)	0.64 (0.61–0.68)	0.721	0.67 (0.61–0.71)	0.63 (0.60–0.67)	0.749
IVIM-DKI_D	1.09 (0.94–1.26)	1.26 (1.17–1.34)	0.045^*^	1.07 (0.92–1.17)	1.26 (1.17–1.34)	0.037^*^
IVIM-DKI_Dp	22.75 (19.91–24.04)	21.69 (19.58–24.37)	0.905	23.46 (20.58–24.57)	21.65 (19.58–24.32)	0.749
IVIM-DKI_f	0.18 (0.17–0.20)	0.19 (0.18–0.21)	0.275	0.19 (0.17–0.20)	0.19 (0.18–0.21)	0.526
IVIM-DKI_K	0.51 (0.42–0.57)	0.56 (0.52–0.60)	0.112	0.51 (0.42–0.59)	0.56 (0.51–0.59)	0.438
CTRW_D	1.17 (1.13–1.29)	1.30 (1.24–1.39)	0.021^*^	1.15 (1.07–1.21)	1.30 (1.24–1.39)	0.028^*^
CTRW_alpha	0.85 (0.81–0.97)	0.92 (0.90–0.94)	0.040^*^	0.81 (0.79–0.91)	0.92 (0.90–0.94)	0.022^*^
CTRW_beta	0.76 (0.71–0.80)	0.75 (0.71–0.79)	0.721	0.74 (0.71–0.82)	0.75 (0.71–0.79)	1.000
FROC_D	1.15 (1.07–1.28)	1.30 (1.24–1.40)	0.016^*^	1.07 (1.04–1.15)	1.30 (1.24–1.40)	0.022^*^
FROC_beta	0.76 (0.72–0.79)	0.75 (0.71–0.78)	0.682	0.73 (0.72–0.79)	0.75 (0.71–0.78)	0.969
FROC_mu	7.51 (7.08–7.70)	7.68 (7.56–7.83)	0.026^*^	7.53 (6.93–7.70)	7.67 (7.54–7.81)	0.186

All diffusion-related parameters are presented as median (interquartile range)

Statistical comparisons were performed using the Mann–Whitney *U*-test, and the false discovery rate (FDR) method was used for multiple comparisons

*p*-values with an asterisk (*) superscript indicate statistical significance

Units: MEM_ADC, IVIM_D, IVIM_Dstar, DKI_D, SEM_DDC, FROC_D, CTRW_D: ×10⁻³ mm²/s; FROC_mu: μm⁻¹; DKI_K, SEM_Alpha, FROC_beta, CTRW_alpha, CTRW_beta, IVIM_f: unitless

### Evaluation of cross-vendor robustness

Following ComBat harmonization, the quantitative distributions of 17 out of 18 diffusion parameters (with the exception of DKI_K) showed no significant differences between the primary and cross-vendor cohorts (all *p* > 0.05; Supplementary Fig. S7), demonstrating the robust cross-protocol consistency of the diffusion models. When applied to this cross-vendor cohort, all three constructed models maintained stable diagnostic performance. The Clinical, Clinical+ADC, and Clinical+Multib_DWI models achieved AUCs of 0.74, 0.84, and 0.88, respectively. Notably, the Clinical+Multib_DWI model demonstrated the highest generalizability and consistently yielded higher net benefits than the other models across threshold probabilities > 0.1 in DCA (Fig. 6g–i).

In the exploratory analysis of the zone-specific split model, the algorithm demonstrated comparable internal validation performance to the integrated model (Test AUC = 0.840). However, its diagnostic efficacy noticeably declined in the multi-vendor validation cohort (External AUC = 0.825), alongside reduced net benefit, indicative of cross-vendor overfitting (Supplementary Table S4 and Fig. S8).

## Discussion

In this study, we systematically evaluated seven multi-*b*-value DWI models and identified MEM_ADC and CTRW_alpha as complementary biomarkers for csPCa detection. Importantly, anatomical location played a pivotal role: MEM_ADC achieved the highest accuracy in PZ, whereas CTRW_alpha provided superior value in TZ, suggesting that zone-specific diffusion application optimizes risk stratification.

The observed zone-specific divergence in model performance reflects histological differences. The PZ is predominantly composed of glandular tissue with uniform acinar structures and less stromal complexity [29], where water diffusion approximates a Gaussian distribution, making MEM_ADC biologically plausible and rendering the advanced complexity profiling of CTRW_alpha redundant. Conversely, the TZ is characterized by a heterogeneous mixture of stromal hyperplasia, dense fibromuscular tissue, and interspersed glands [30]. Such complex gland-stroma interactions create multiple diffusion barriers and microstructural compartments, leading to markedly non-Gaussian diffusion behavior [31]. The CTRW model, specifically the alpha parameter, characterizes the temporal heterogeneity of water diffusion. In the dense cellularity and disordered stroma of csPCa, water molecules exhibit anomalous subdiffusion, frequently becoming “trapped” by organelles and irregular membranes [12]. A lower alpha value indicates a longer “waiting time” within these compartments. CTRW_alpha thereby provides a more sensitive probe for detecting malignancy amidst the stromal background of TZ.

Our findings align with the limited literature on CTRW in PCa [32] and other malignancies [15, 17, 33–36]. These studies consistently identified alpha as a stable predictor of malignancy, suggesting that CTRW_alpha captures universal features of tumor cytoarchitecture, validating its utility in the prostate TZ. In contrast, FROC_beta showed diagnostic value only in the PZ subgroup, contradicting some prior reports [37, 38], suggesting its sensitivity may also be spatially dependent.

Regarding model construction, the Clinical+Multib_DWI model did not statistically improve AUC over the other models in the test and validation sets. The relatively higher discriminative power of ADC in the PZ limited the incremental value of CTRW_alpha in that subgroup. In contrast, CTRW_alpha contributed more substantially in TZ, where ADC alone performed less effectively. This aligns with the PI-RADS v2.1 framework, which prioritizes T2WI over DWI/ADC for TZ lesions [4]. However, while the overall statistical improvement in AUC was marginal, decision curve analysis highlighted the superior clinical utility of the multi-*b*-value model. Specifically, it provided a consistently higher net benefit within the 0.1–0.4 threshold probability range. In routine practice, this 10%–40% risk interval represents a critical diagnostic “gray zone” where physicians constantly struggle to balance the vital detection of csPCa against unnecessary biopsies [39]. By optimizing the diagnostic trade-off specifically within this equivocal window, the integrated model enables more confident triage—safely sparing more patients from invasive procedures without compromising cancer detection.

Technically, non-Gaussian models like CTRW require high *b*-value data ( > 2000 s/mm^2^) [40], which is typically prone to geometric distortion and susceptibility artifacts [41]. To address this, we employed the ZOOMit sequence (rFOV DWI) in our primary cohort. This technique provides superior contrast and resolution, ensuring that the analyzed signal decay reflects true microstructural restrictions rather than image artifacts. Notably, our cross-vendor validation was successfully performed on a GE scanner using a standard full-FOV DWI sequence. The consistent diagnostic performance observed in this heterogeneous cohort suggests that the microstructural biomarkers identified using high-quality ZOOMit data are robust and transferable to routine clinical settings, even when specialized rFOV sequences are unavailable.

Finally, we evaluated an exploratory unified zone-aware model (conditionally applying MEM_ADC for PZ and CTRW_alpha for TZ). While this split-cohort model demonstrated excellent internal performance, its generalizability slightly decreased in the multi-vendor validation cohort compared to our integrated model. This attenuation is likely due to the reduced sample size within each anatomical sub-cohort, which compromises the statistical power required to robustly weight shared clinical covariates, leading to cross-vendor overfitting. Therefore, while building a conditionally-switching zone-aware algorithm remains the ultimate goal for future large-scale multicenter studies, our current integrated model represents the most statistically robust and pragmatic unified workflow for current clinical applications.

This study has limitations. First, the reference standard relied on biopsy rather than whole-mount pathology. In the historical validation cohort with only systematic biopsy, radiological-pathological correlation via regional spatial matching carries an inherent risk of spatial misalignment, requiring future validation with whole-mount step-section pathology for precise co-registration. Second, the limited sample size in subgroup analyses—particularly csPCa cases within TZ PI-RADS 3 lesions (*n* = 9)—may constrain statistical power, warranting validation in larger multi-center cohorts. Third, manual segmentation and reliance on mean parameter metrics may limit efficiency and overlook intralesional variability. Incorporating automatic segmentation and histogram-based features could better capture PCa heterogeneity. Finally, technical and workflow barriers remain. Acquiring multiple high *b*-values for non-Gaussian models prolongs scan time, potentially increasing motion artifacts and reducing clinical throughput. Additionally, offline post-processing poses challenges to routine implementation. Future protocol optimization (e.g., identifying the minimum required *b*-values) and the integration of AI-assisted commercial software will be essential to facilitate the clinical translation of these advanced metrics.

## Conclusion

MEM_ADC and CTRW_alpha are independent predictors of csPCa. Although the combined model showed no statistically significant improvement in AUC over the Clinical+ADC model, it yielded a superior NPV. Importantly, the relative value of diffusion metrics was zone-dependent: MEM_ADC was most informative in PZ, whereas CTRW_alpha played a greater role in TZ, suggesting that location-specific application of diffusion models may be necessary in clinical practice.

## Supplementary information

ELECTRONIC SUPPLEMENTARY MATERIAL

## Data Availability

The datasets generated or analyzed during the study are not publicly available due to institutional privacy restrictions, but are available from the corresponding author on reasonable request.

## Abbreviations

ADC: Apparent diffusion coefficient
AUC: Areas under the curve
csPCa: Clinically significant prostate cancer
CTRW: Continuous-time random walk
DKI: Diffusion kurtosis imaging
DRE: Digital rectal examination
DWI: Diffusion-weighted imaging
FROC: Fractional order calculus
GG: Gleason grade
IVIM: Intravoxel incoherent motion
OR: Odds ratio
PI-RADS: Prostate Imaging-Reporting and Data System
PSA: Prostate-specific antigen
PSAD: Prostate-specific antigen density
PZ: Peripheral zone
ROC: Receiver operating characteristic
ROI: Region of interest
SEM: Stretched exponential model
TZ: Transition zone
