# Supplementary material for: Precise zonal diagnosis: multi-b-value DWI model reveals differential predictors of clinically significant prostate cancer in peripheral and transition zones
Source: Insights Imaging. 2026 Jun 5;17:154. doi: 10.1186/s13244-026-02324-2 (PMC13241545; doi:10.1186/s13244-026-02324-2)
Supplement: Supplementary file 1 — ELECTRONIC SUPPLEMENTARY MATERIAL [file 13244_2026_2324_MOESM1_ESM.pdf]

Precise zonal diagnosis: multi-b-value DWI model reveals differential predictors of clinically significant prostate cancer in peripheral and transition zones

ELECTRONIC SUPPLEMENTARY MATERIAL

Supplementary Methods

1. MpMRI protocols

Manufacturer	Sequences	Type	TR/TE (ms)	Slice thickness (mm)	Gap (mm)	FOV (mm^2)	Matrix	Voxel size (mm^3)	NEX	Flip angle	Time
Siemens	T1WI_Tra	TSE	807/13	5	0	300*357	396*396	0.5*0.5*5	1	141°	1m40s
	T2WI_Tra	TSE	5800/139	3	0	180*180	360*360	0.5*0.5*3	2	160°	2m48s
	Zoomit-DWI	EPI	4600/94	3	0	260*113	236*102	1.1*1.1*3	/	90°	8m31s
GE	T1WI_Tra	TSE	546/8.2	5	0	360*360	512*512	0.7*0.7*5	1	111°	3m03s
	T2WI_Tra	TSE	3350/120	3	0	180*180	512*512	0.35*0.35*3	1	111°	3m10s
	Standard DWI	EPI	3000/80	3	0	280*280	256*256	1.09*1.09*3	/	90°	6m55s

T1-weighted imaging (T1WI), T2-weighted imaging (T2WI), repetition time (TR)/echo time (TE), field of view (FOV), number of excitations (NEX), transverse (Tra), sagittal (Sag), coronal (Cor), fat suppression (Fs), Turbo Spin Echo (TSE).

The b-values for Siemens MR scanner: 0_1, 20_1, 50_1, 80_1, 100_1, 200_1, 500_1, 800_1, 1000_3, 1500_4, 2000_5, and 3000_8 s/mm², and for GE MR scanner: 0_1, 10_2, 25_2, 50_2, 75_2, 100_2, 150_2, 200_2, 500_2, 1000_4, 1500_4, 2000_6, 2500_6, and 3000_6 s/mm² (subscripts indicate the number of signal averages).

2. Diffusion model fitting methods

2.1 The mono-exponential model was fitted according to Equation (2-1):

$$\frac{S}{S_0} = e^{-b \cdot ADC} \tag{2-1}$$

This model is the simplest diffusion model, in which at least two b-values are required to estimate the apparent diffusion coefficient (ADC). In this study, the ADC map was calculated using four b-values. In the equation, S_0 and S represent the voxel signal intensities at b=200, 500, and b=1000 s/mm^2, respectively; b denotes the diffusion weighting factor, and ADC represents the apparent diffusion coefficient. The resulting parameter map was denoted as MEM_ADC.

2.2 The IVIM model was fitted according to Equation (2-2):

$$\frac{S}{S_0} = (1 - f) \cdot e^{(-b \cdot D)} + f \cdot e^{(-b \cdot D*)} \tag{2-2}$$

This model was first proposed by Le Bihan et al. in 1986. It incorporates the influence of microvascular perfusion on ADC estimation. At low b-values (0–200 s/mm^2), the diffusion signal is determined not only by the diffusion of water molecules in tissue but also by the incoherent microcirculation within the capillary network. Therefore, a sufficient number of low b-values is required to disentangle the perfusion contribution and to estimate three parameters: the true diffusion coefficient (D), the pseudo-diffusion coefficient (D*), and the perfusion fraction (f).

In this study, the IVIM model was fitted using b-values of 0, 20, 50, 80, 100, 200, 500, 800, and 1000 s/mm^2 for Siemens data and 0, 25, 50, 75, 100, 150, 200, 500, 1000 s/mm^2 for GE data. In the equation, S denotes the signal intensity at a given b-value, S_0 represents the signal intensity at b=0 s/mm^2, D is the true diffusion coefficient, $D*$ (Dstar) is the pseudo-diffusion coefficient, and f is the perfusion fraction. The resulting parameter maps were denoted as IVIM_D, IVIM_Dstar, and IVIM_f.

2.3 The DKI model was fitted according to Equation (2-3):

$$\frac{S}{S_0} = e^{(-b \cdot D + \frac{b^2 \cdot D^2 \cdot K}{6})} \quad (2\text{-}3)$$

This model was proposed by Jensen et al. in 2005 to estimate diffusion kurtosis at higher b-values, thereby quantifying the non-Gaussian distribution of water diffusion. It introduces two parameters: the apparent kurtosis coefficient (K), which reflects the degree to which diffusion deviates from a Gaussian distribution, and the apparent diffusion coefficient (D), which represents the diffusion coefficient corrected for non-Gaussian behavior.
In this study, the DKI model was fitted using b-values of 0, 200, 500, 1000, 1500, 2000, and 3000 s/mm². The resulting parameter maps were denoted as DKI_D and DKI_K.

2.4 The SEM was fitted according to Equation (2-4):

$$\frac{S}{S_0} = e^{(-b \cdot DDC)^{\alpha}} \quad (2\text{-}4)$$

The SEM was introduced by Bennett in 2003 to characterize intravoxel heterogeneity of diffusion rates (heterogeneity index, α\alpha) and distributed diffusion effects (distributed diffusion coefficient, DDC). The DDC reflects the overall rate of signal decay across different b-values, while α\alpha ranges between 0 and 1, indicating the deviation from the mono-exponential model. In the equation, S denotes the signal intensity at a given b-value, and S_0 represents the signal intensity at b=0 s/mm². In this study, the SEM model was fitted using the entire spectrum of acquired b-values. The resulting parameter maps were denoted as SEM_alpha and SEM_DDC.

2.5 The FROC model was fitted according to Equation (2-5):

$$\frac{S}{S_0} = e^{(-(b \cdot D)^{\beta} + \mu \cdot b)} \quad (2\text{-}5)$$

The FROC model, derived from fractional-order calculus theory, was introduced to describe anomalous diffusion processes that deviate from the conventional Gaussian assumption. It characterizes tissue microstructural complexity by incorporating three parameters: the diffusion coefficient (D), the fractional order parameter (β\beta), and the spatial correlation length (μ\mu). Specifically, β\beta reflects the degree of deviation from Gaussian diffusion, while μ\mu accounts for spatial structural correlations within tissue. In the equation, S and S_0 represent the signal intensities at a given b-value and at b=0, respectively. In this study, the FROC model was fitted using the entire spectrum of acquired b-values. The resulting parameter maps were denoted as FROC_D, FROC_beta, and FROC_mu.

2.6 The CTRW model was fitted according to Equation (2-6):

$$\frac{S}{S_0} = E\alpha^{(-(b \cdot Dm)^{\beta})} \quad (2\text{-}6)$$

The CTRW model, based on stochastic diffusion theory, was proposed to capture non-Gaussian water diffusion behavior by modeling the anomalous diffusion process as a continuous-time random walk. This model introduces three key parameters: the anomalous diffusion coefficient (Dm), the temporal diffusion heterogeneity index (α\alpha), and the spatial heterogeneity index (β\beta). Here, α\alpha describes temporal complexity of diffusion, β\beta reflects spatial heterogeneity, and Dm represents the generalized diffusion coefficient. In the equation, Eα denotes the Mittag–Leffler function, S is the signal intensity at a given b-value, and S0 is the signal intensity at b=0. In this study, the CTRW model was fitted using the entire spectrum of acquired b-values. The resulting parameter maps were denoted as CTRW_Dm, CTRW_alpha, and CTRW_beta.

2.7 The hybrid IVIM-DKI model was fitted according to Equation (2-7):

$$\frac{S}{S_0} = (1-f)e^{\left(-b \cdot D + \frac{b^2 \cdot D^2 \cdot K}{6}\right)} + fe^{-bD^*} \quad (2\text{-}7)$$

The IVIM-DKI model integrates the intravoxel incoherent motion (IVIM) framework with diffusion kurtosis imaging (DKI), thereby accounting for both microvascular perfusion and non-Gaussian diffusion effects. It allows simultaneous estimation of perfusion-related and kurtosis-related diffusion parameters, improving sensitivity to tissue microstructural alterations. The model provides four parameters: the true diffusion coefficient (D), the kurtosis coefficient (K), the pseudo-diffusion coefficient (D∗), and the perfusion fraction (f). In the equation, S and S_0 represent signal intensities at a given b-value and at b=0, respectively. In this study, the IVIM-DKI model was fitted using the entire spectrum of acquired b-values. The resulting parameter maps were denoted as IVIM-DKI_D, IVIM-DKI_K, IVIM-DKI_Dstar, and IVIM-DKI_f.

2.8 All derived diffusion parameter list: MEM_ADC, SEM_DDC, SEM_alpha, IVIM_D, IVIM_Dstar, IVIM_f, DKI_D, DKI_K, IVIM-DKI_D, IVIM-DKI_Dp, IVIM-DKI_f, IVIM-DKI_K, CTRW_D, CTRW_alpha, CTRW_beta, FROC_D, FROC_beta, and FROC_mu.

3. Supplementary Methods: Construction and Evaluation of the Exploratory Unified Zone-Aware Model

3.1 Purpose: Prompted by the observed spatial complementarity of diffusion metrics, we conducted an exploratory analysis to evaluate the theoretical potential of a strictly location-dependent predictive workflow (i.e., exclusively applying standard ADC for peripheral zone [PZ] lesions and advanced CTRW_alpha for transition zone [TZ] lesions). The objective was to determine whether mechanically stratifying the dataset to build zone-specific sub-models could yield a unified probability score that outperforms our primary integrated model (Clinical + Multib_DWI) and to assess its cross-vendor generalizability within the constraints of our current sample size.

3.2 Method

The construction of this exploratory unified zone-aware model consisted of three main steps: cohort stratification, zone-specific sub-model training, and unified conditional validation.

Cohort Stratification and Sub-model Training: Instead of analyzing the entire training cohort collectively, the training set was anatomically stratified into two distinct sub-cohorts: a PZ subset (n = 69) and a TZ subset (n =88). Standard multivariable logistic regression was utilized for the sub-model. For the PZ sub-model, only standard clinical variables (age, PSA density, digital rectal examination status, prostate volume, and PI-RADS scores) and the MEM_ADC value were incorporated as predictors. For the TZ sub-model, the same standard clinical variables were used, paired exclusively with the CTRW_alpha value. Continuous variables were standardized using Z-score normalization prior to modeling to prevent mathematical overflow during algorithm fitting.

Unified Conditional Validation: To simulate a practical, unified clinical workflow for the validation cohorts (the internal test set and the external GE cross-vendor set), an algorithmic wrapper was developed. This conditional algorithm first identified the anatomical location of each lesion in the validation sets. It then dynamically routed the lesion data to the corresponding pre-trained sub-model: the PZ sub-model was applied to generate a predicted probability for PZ lesions, while the TZ sub-model was applied for TZ lesions.

Overall Evaluation: The conditionally predicted probabilities from both anatomical zones were then concatenated into a single, unified probability array for each validation cohort. This allowed for the calculation of overall diagnostic metrics—including the area under the receiver operating characteristic curve (AUC) and decision curve analysis (DCA)—across the entire validation cohort, enabling a direct and fair comparison with the primary models evaluated in the main manuscript.

3.3 Result The results of the construction and evaluation of this Exploratory Unified Zone-Aware Model were shown in Supplementary Table S4 and Figure S8.

Supplementary Tables

Table S1. Interobserver Agreement of Diffusion Parameters Assessed by Intraclass Correlation Coefficient (ICC)

Parameters	ICC
**MEM_ADC**	0.92
**SEM_DDC**	0.89
**SEM_alpha**	0.85
**IVIM_D**	0.94
**IVIM_Dstar**	0.84
**IVIM_f**	0.83
**DKI_D**	0.94
**DKI_K**	0.98
**IVIM-DKI_D**	0.94
**IVIM-DKI_Dp**	0.84
**IVIM-DKI_f**	0.82
**IVIM-DKI_K**	0.89
**CTRW_D**	0.91
**CTRW_alpha**	0.91
**CTRW_beta**	0.84
**FROC_D**	0.94
**FROC_beta**	0.87
**FROC_mu**	0.89

Table S2. Comparison of the radiographic characteristics and DWI model parameters for distinguishing csPCa in PI-RADS category 3 or category 4-5 lesions.

	Category 3			Category 4-5		
MRI Parameters	csPCa （n =28）	Non- csPCa （n = 90）	*p*	csPCa （n =87）	Non- csPCa （n = 19）	*p*
Lesion diameter	1.34 (0.91-1.63)	1.60 (1.33-1.98)	0.039*	2.80 (2.08-3.89)	1.65 (1.36-2.61)	0.003*
Lesion volume	0.45 (0.26-0.78)	1.03 (0.65-1.94)	0.001*	4.04 (2.11-10.96)	1.44 (0.94-3.63)	0.004*
MEM_ADC	0.99 (0.94-1.08)	1.13 (1.05-1.20)	0.001*	0.82 (0.76-0.90)	1.00 (0.82-1.09)	0.004*
SEM_DDC	1.27 (1.12-1.43)	1.39 (1.31-1.51)	0.021*	0.95 (0.87-1.09)	1.21 (1.06-1.41)	0.003*
SEM_alpha	0.70 (0.65-0.72)	0.71 (0.68-0.74)	0.104	0.69 (0.65-0.70)	0.69 (0.66-0.71)	0.856
IVIM_D	0.94 (0.83-1.04)	1.05 (0.96-1.11)	0.021*	0.77 (0.71-0.82)	0.93 (0.74-1.01)	0.012*
IVIM_Dstar	12.20 (9.68-17.74)	15.05 (12.41-18.90)	0.053	13.61 (11.88-15.76)	13.92 (11.89-16.21)	0.723
IVIM_f	0.17 (0.13-0.21)	0.20 (0.17-0.23)	0.045*	0.16 (0.14-0.18)	0.18 (0.16-0.20)	0.063
DKI_D	1.34 (1.21-1.50)	1.53 (1.45-1.65)	0.001*	1.11 (0.99-1.23)	1.33 (1.21-1.51)	0.003*
DKI_K	0.66 (0.57-0.71)	0.63 (0.59-0.67)	0.471	0.76 (0.69-0.81)	0.67 (0.65-0.70)	0.012*
IVIM-DKI_D	1.12 (1.05-1.26)	1.26 (1.20-1.34)	0.002*	0.89 (0.81-0.98)	1.08 (0.89-1.22)	0.004*
IVIM-DKI_Dp	20.81 (16.33-24.17)	21.39 (18.18-23.96)	0.537	20.93 (18.94-23.40)	22.01 (17.75-23.78)	0.755
IVIM-DKI_f	0.20 (0.17-0.23)	0.19 (0.18-0.21)	0.473	0.18 (0.17-0.20)	0.19 (0.17-0.21)	0.355
IVIM-DKI_K	0.51 (0.43-0.58)	0.55 (0.46-0.59)	0.356	0.60 (0.53-0.67)	0.56 (0.52-0.61)	0.112
CTRW_D	1.18 (1.11-1.33)	1.31 (1.24-1.41)	0.002*	0.95 (0.89-1.05)	1.14 (1.04-1.30)	0.003*
CTRW_alpha	0.87 (0.81-0.92)	0.92 (0.90-0.94)	0.001*	0.83 (0.79-0.87)	0.90 (0.87-0.93)	<0.001*
CTRW_beta	0.77 (0.72-0.83)	0.76 (0.71-0.80)	0.399	0.76 (0.72-0.79)	0.76 (0.72-0.77)	0.549
FROC_D	1.15 (1.07-1.27)	1.30 (1.24-1.40)	0.001*	0.95 (0.87-1.07)	1.13 (1.06-1.27)	0.003*
FROC_beta	0.77 (0.73-0.82)	0.76 (0.71-0.79)	0.401	0.74 (0.71-0.76)	0.75 (0.72-0.76)	0.644
FROC_mu	7.54 (7.34-7.72)	7.67 (7.52-7.83)	0.041*	7.34 (7.22-7.54)	7.48 (7.33-7.63)	0.145

Table S3. Multivariate Logistic Regression Analysis of Clinical and Diffusion Parameters for Predicting PCa and csPCa in PI-RADS 3 lesions in TZ

	PCa				csPCa			
**Variables**	**Coefficient**	**OR (95%CI)**	**Std_Error**	***p*-value**	**Coefficient**	**OR (95%CI)**	**Std_Error**	***p*-value**
**Age**	0.004	1.004 (0.917-1.099)	0.046	0.935	0.019	1.019 (0.860-1.207)	0.086	0.828
**PSAD**	0.663	1.940 (0.027-141.368)	2.188	0.762	8.885	25.284 (1.751-298.562)	4.248	0.036*
**DRE**	-0.398	0.671 (0.174-2.586)	0.688	0.563	-3.855	0.021 (0.000-1.173)	2.049	0.060
**Lesion_volume**	-0.074	0.929 (0.658-1.311)	0.176	0.674	-0.138	0.872 (0.462-1.784)	0.365	0.707
**CTRW_alpha**	-18.421	0.000 (0.000-0.008)	6.953	0.008*	-41.522	0.000 (0.000-0.000)	14.222	0.004*
**MEM_ADC**	-5.965	0.003 (0.000-0.648)	2.822	0.035*	-9.038	0.000 (0.000-1.803)	4.912	0.066

Table S4. Comparison of the exploratory Unified_Zone_Aware model with other three models in the training, test, and cross-vendor set

		AUC	AUC_CI	Threshold	Accuracy	Sensitivity	Specificity	PPV	NPV
Train	Clinical	0.893	0.836-0.938	0.659	0.828	0.700	0.961	0.949	0.755
	Clinical+ADC	0.932	0.886-0.966	0.373	0.873	0.888	0.857	0.866	0.880
	Clinical+Multib_DWI	0.945	0.905-0.973	0.250	0.847	0.938	0.753	0.798	0.921
	Unified_Zone_Aware	0.943	0.903-0.974	0.204	0.873	0.988	0.753	0.806	0.983
Test	Clinical	0.782	0.668-0.882	0.271	0.746	0.857	0.625	0.714	0.800
	Clinical+ADC	0.797	0.685-0.892	0.421	0.776	0.800	0.750	0.778	0.774
	Clinical+Multib_DWI	0.846	0.749-0.931	0.250	0.806	0.914	0.688	0.762	0.880
	Unified_Zone_Aware	0.840	0.727-0.935	0.425	0.851	0.886	0.813	0.838	0.867
Cross-Vendor	Clinical	0.735	0.626-0.832	0.250	0.702	0.973	0.489	0.600	0.958
	Clinical+ADC	0.836	0.738-0.917	0.371	0.810	0.946	0.702	0.714	0.943
	Clinical+Multib_DWI	0.876	0.790-0.947	0.250	0.821	0.973	0.702	0.720	0.971
	Unified_Zone_Aware	0.825	0.732-0.905	0.246	0.774	0.946	0.638	0.673	0.938

Supplementary Figures

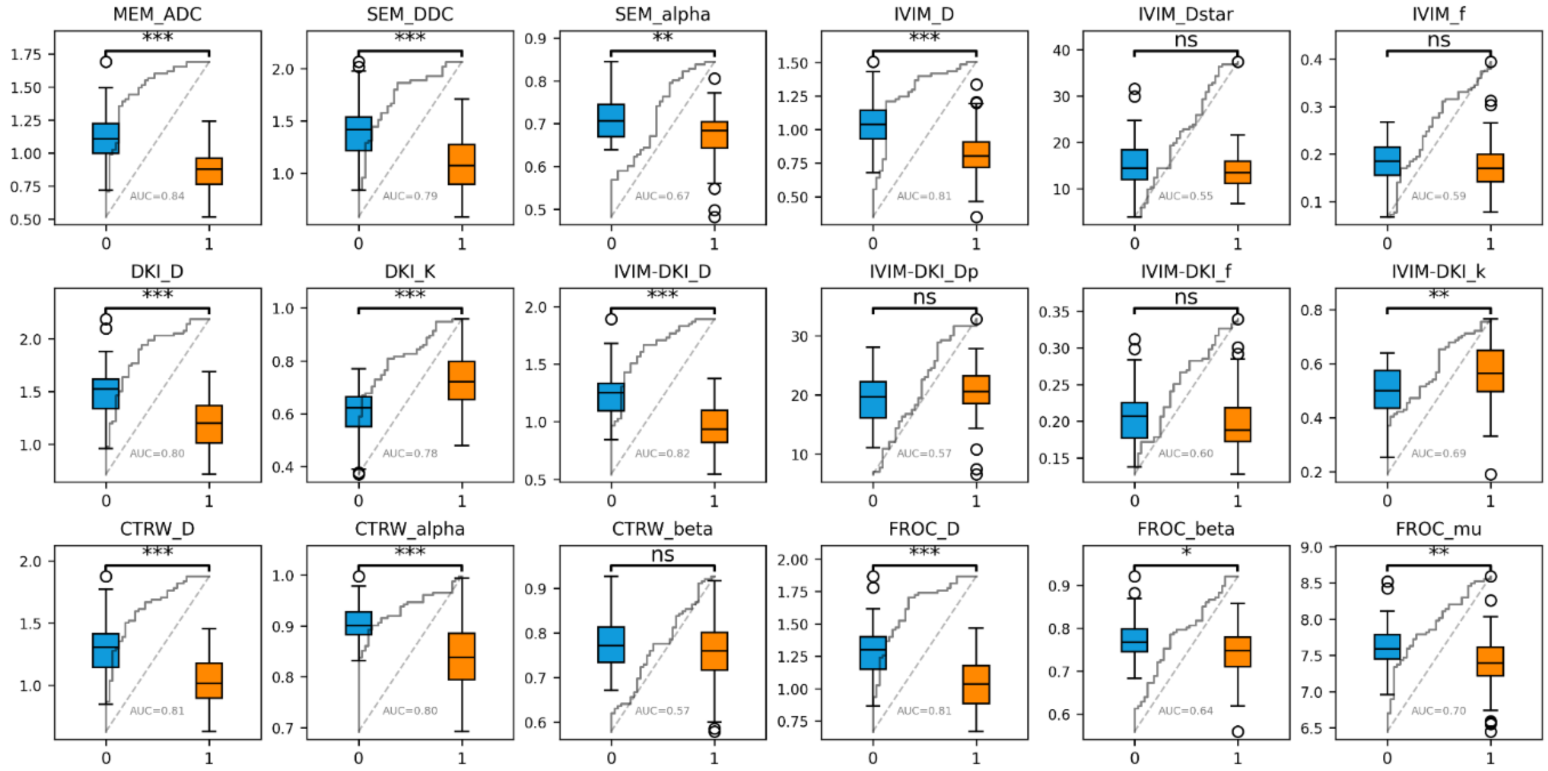

Figure S1. Boxplots with embedded ROC curves of DWI parameters compared in csPCa (1) and non-csPCa (0) in PZ subgroup.

Units: Mono_ADC, SEM_DDC, IVIM_D, IVIM_Dstar, DKI_D, IVIM_DKI_D, IVIM_DKI_Dp, CTRW_D, and FROC_D are expressed in $\times 10^{-3}$ mm²/s; FROC_μ in μm; SEM_Alpha, IVIM_f, DKI_K, IVIM-DKI_f, IVIM-DKI_k, FROC_β, CTRW_α, and CTRW_β are dimensionless. AUC: aera under the curve.

‘ns’,‘*’, ‘**’, and ‘***’ indicate FDR adjusted *p*-values ≥0.05, < 0.05, < 0.01, and < 0.001, respectively.

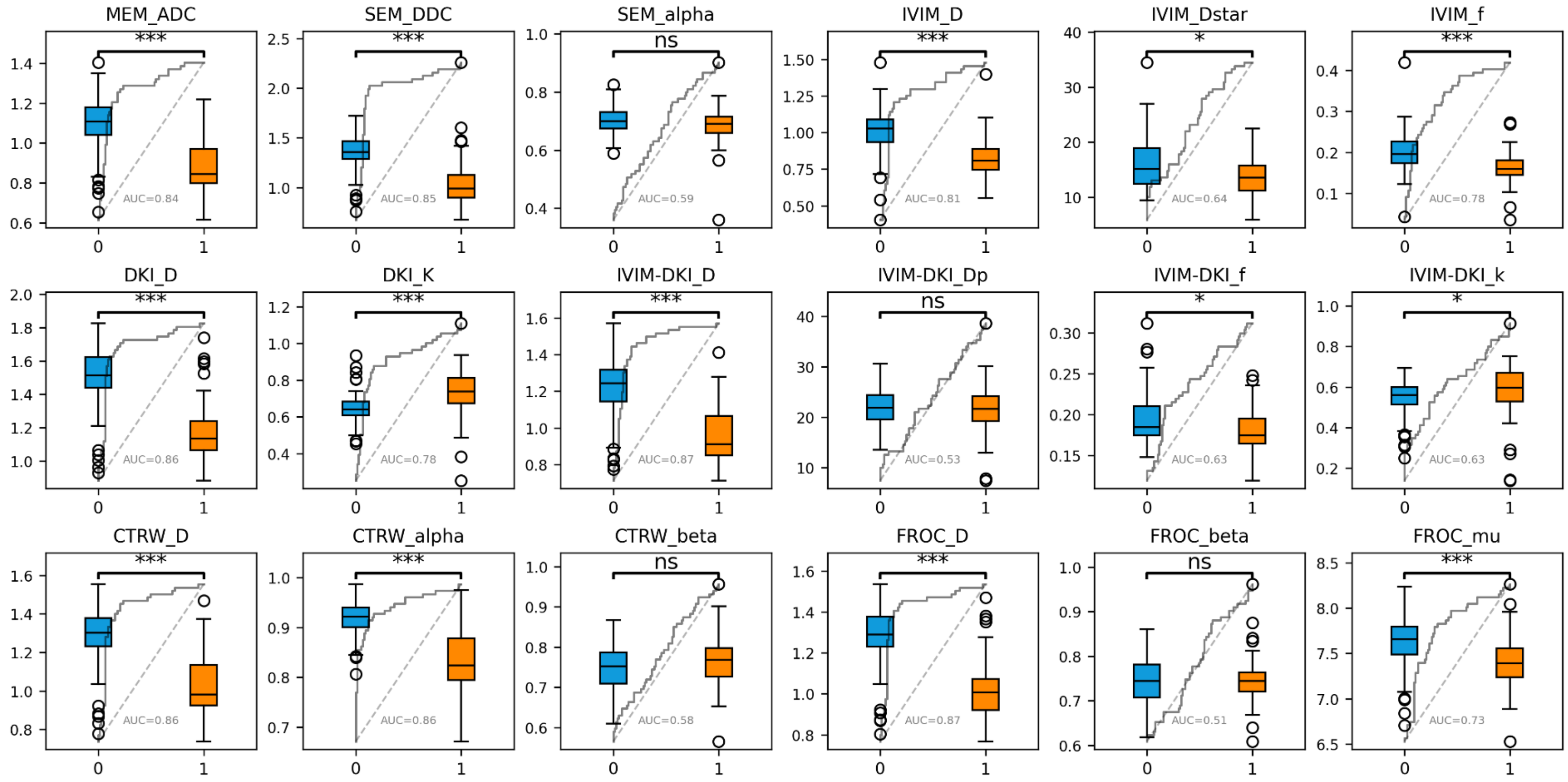

Figure S2. Boxplots with embedded ROC curves of DWI parameters compared in csPCa (1) and non-csPCa (0) in TZ subgroup.
Units: Mono_ADC, SEM_DDC, IVIM_D, IVIM_Dstar, DKI_D, IVIM_DKI_D, IVIM_DKI_Dp, CTRW_D, and FROC_D are expressed in $\times 10^{-3}$ mm²/s; FROC_μ in μm; SEM_Alpha, IVIM_f, DKI_K, IVIM-DKI_f, IVIM-DKI_k, FROC_β, CTRW_α, and CTRW_β are dimensionless. AUC: aera under the curve.
'ns','*', '**', and '***' indicate FDR adjusted *p*-values ≥0.05, < 0.05, < 0.01, and < 0.001, respectively.

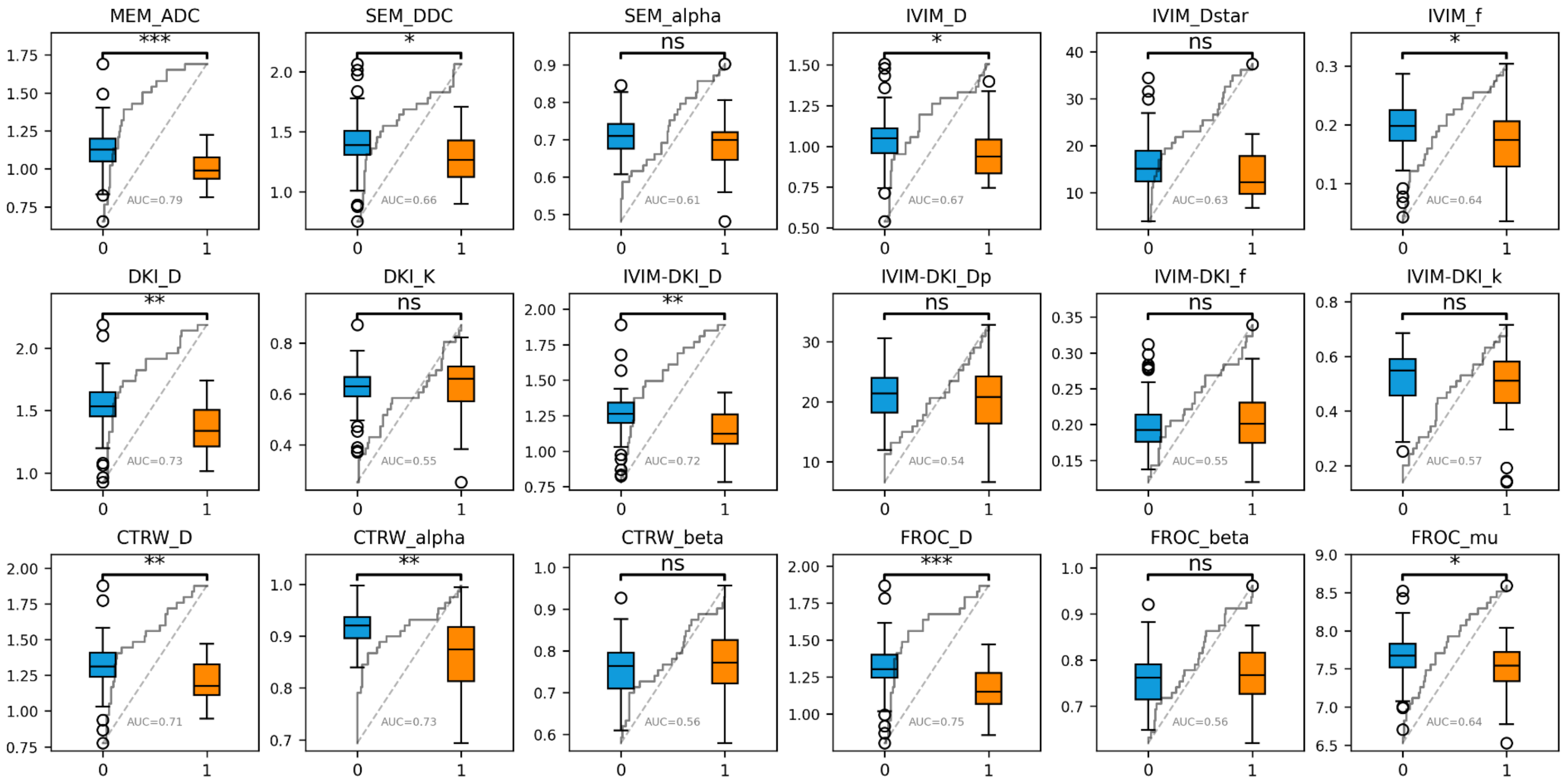

Figure S3. Boxplots with embedded ROC curves of DWI parameters compared in csPCa (1) and non-csPCa (0) in PI-RADS 3 subgroup.
Units: Mono_ADC, SEM_DDC, IVIM_D, IVIM_Dstar, DKI_D, IVIM_DKI_D, IVIM_DKI_Dp, CTRW_D, and FROC_D are expressed in $\times 10^{-3}$ mm²/s; FROC_μ in μm; SEM_Alpha, IVIM_f, DKI_K, IVIM-DKI_f, IVIM-DKI_k, FROC_β, CTRW_α, and CTRW_β are dimensionless. AUC: aera under the curve.
'ns','*', '**', and '***' indicate FDR adjusted *p*-values ≥0.05, < 0.05, < 0.01, and < 0.001, respectively.

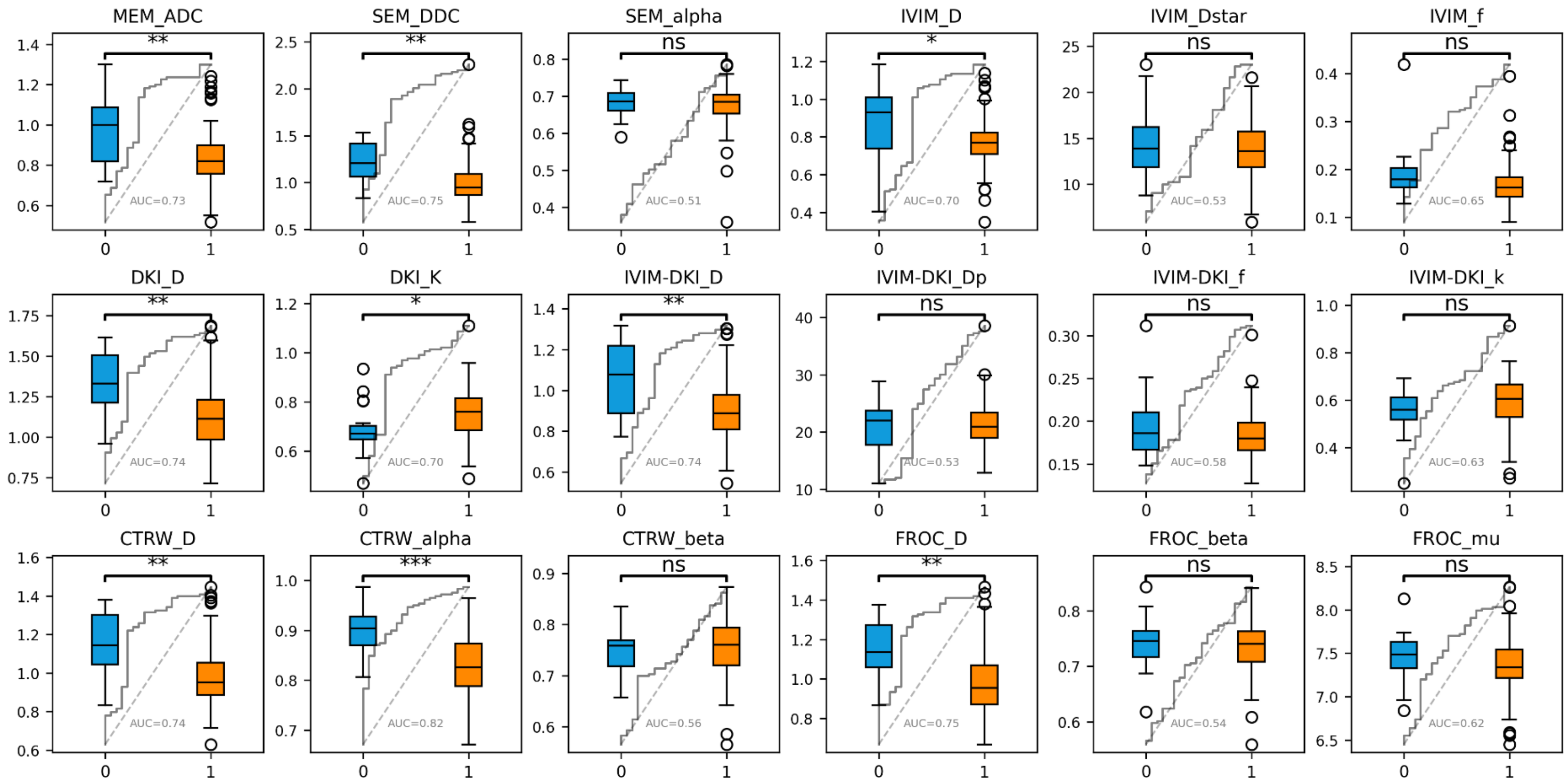

Figure S4. Boxplots with embedded ROC curves of DWI parameters compared in csPCa (1) and non-csPCa (0) in PI-RADS 4–5 subgroup.

Units: Mono_ADC, SEM_DDC, IVIM_D, IVIM_Dstar, DKI_D, IVIM_DKI_D, IVIM_DKI_Dp, CTRW_D, and FROC_D are expressed in $\times 10^{-3}$ mm²/s; FROC_μ in μm; SEM_Alpha, IVIM_f, DKI_K, IVIM-DKI_f, IVIM-DKI_k, FROC_β, CTRW_α, and CTRW_β are dimensionless. AUC: aera under the curve.

'ns','*', '**', and '***' indicate FDR adjusted *p*-values ≥0.05, < 0.05, < 0.01, and < 0.001, respectively.

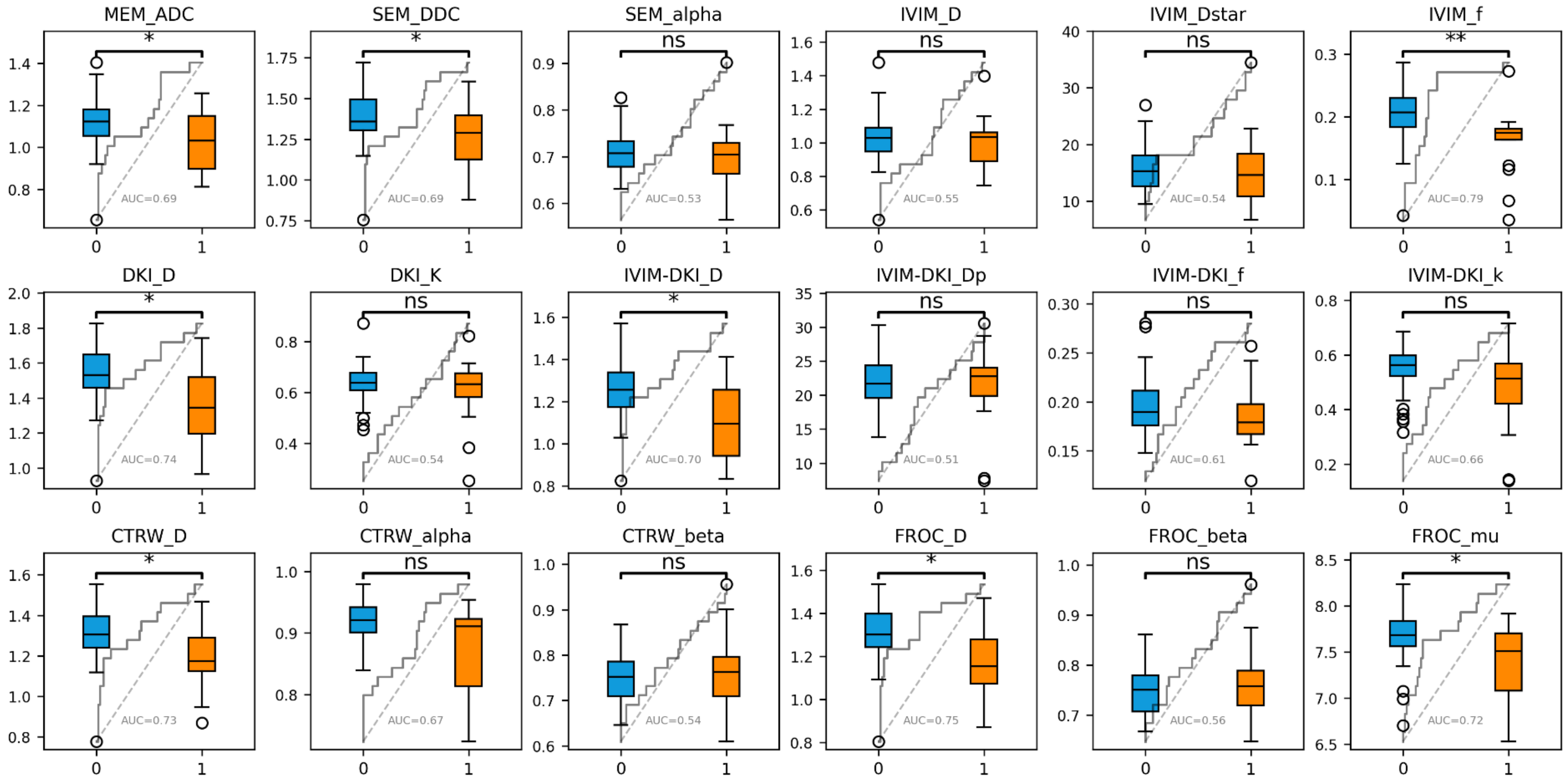

Figure S5. Boxplots with embedded ROC curves of DWI parameters compared in PCa (1) and non-PCa (0) in PI-RADS 3 lesions located in TZ subgroup.

Units: Mono_ADC, SEM_DDC, IVIM_D, IVIM_Dstar, DKI_D, IVIM_DKI_D, IVIM_DKI_Dp, CTRW_D, and FROC_D are expressed in $\times 10^{-3}$ mm^2/s; FROC_μ in μm; SEM_Alpha, IVIM_f, DKI_K, IVIM-DKI_f, IVIM-DKI_k, FROC_β, CTRW_α, and CTRW_β are dimensionless. AUC: aera under the curve.

‘ns’,‘*’, ‘**’, and ‘***’ indicate FDR adjusted *p*-values ≥0.05, < 0.05, < 0.01, and < 0.001, respectively.

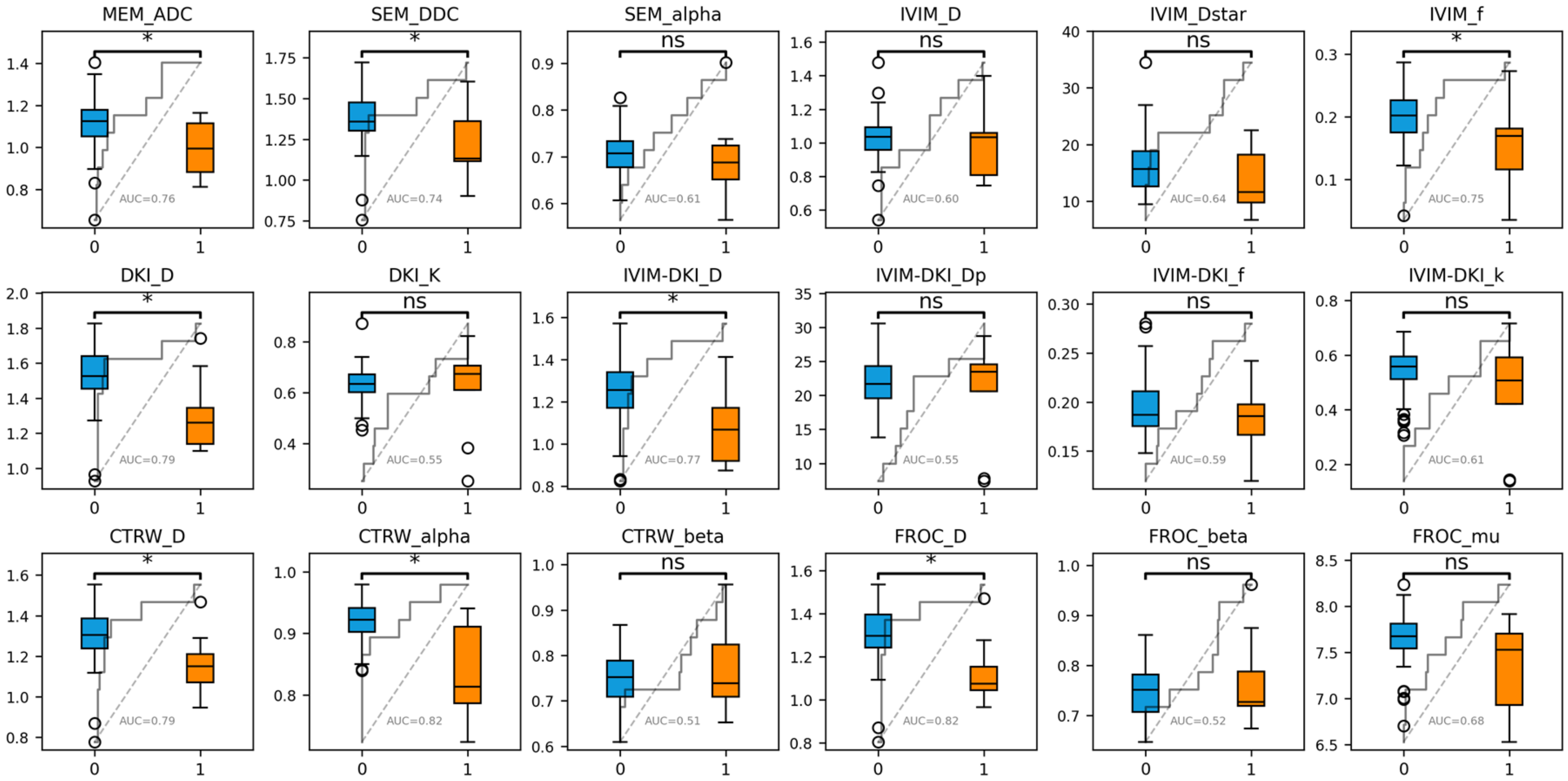

Figure S6. Boxplots with embedded ROC curves of DWI parameters compared in csPCa (1) and non-csPCa (0) in PI-RADS 3 lesions located in TZ subgroup.

Units: Mono_ADC, SEM_DDC, IVIM_D, IVIM_Dstar, DKI_D, IVIM_DKI_D, IVIM_DKI_Dp, CTRW_D, and FROC_D are expressed in $\times 10^{-3}$ mm²/s; FROC_μ in μm; SEM_Alpha, IVIM_f, DKI_K, IVIM-DKI_f, IVIM-DKI_k, FROC_β, CTRW_α, and CTRW_β are dimensionless. AUC: aera under the curve.

'ns','*', '**', and '***' indicate FDR adjusted *p*-values ≥0.05, < 0.05, < 0.01, and < 0.001, respectively.

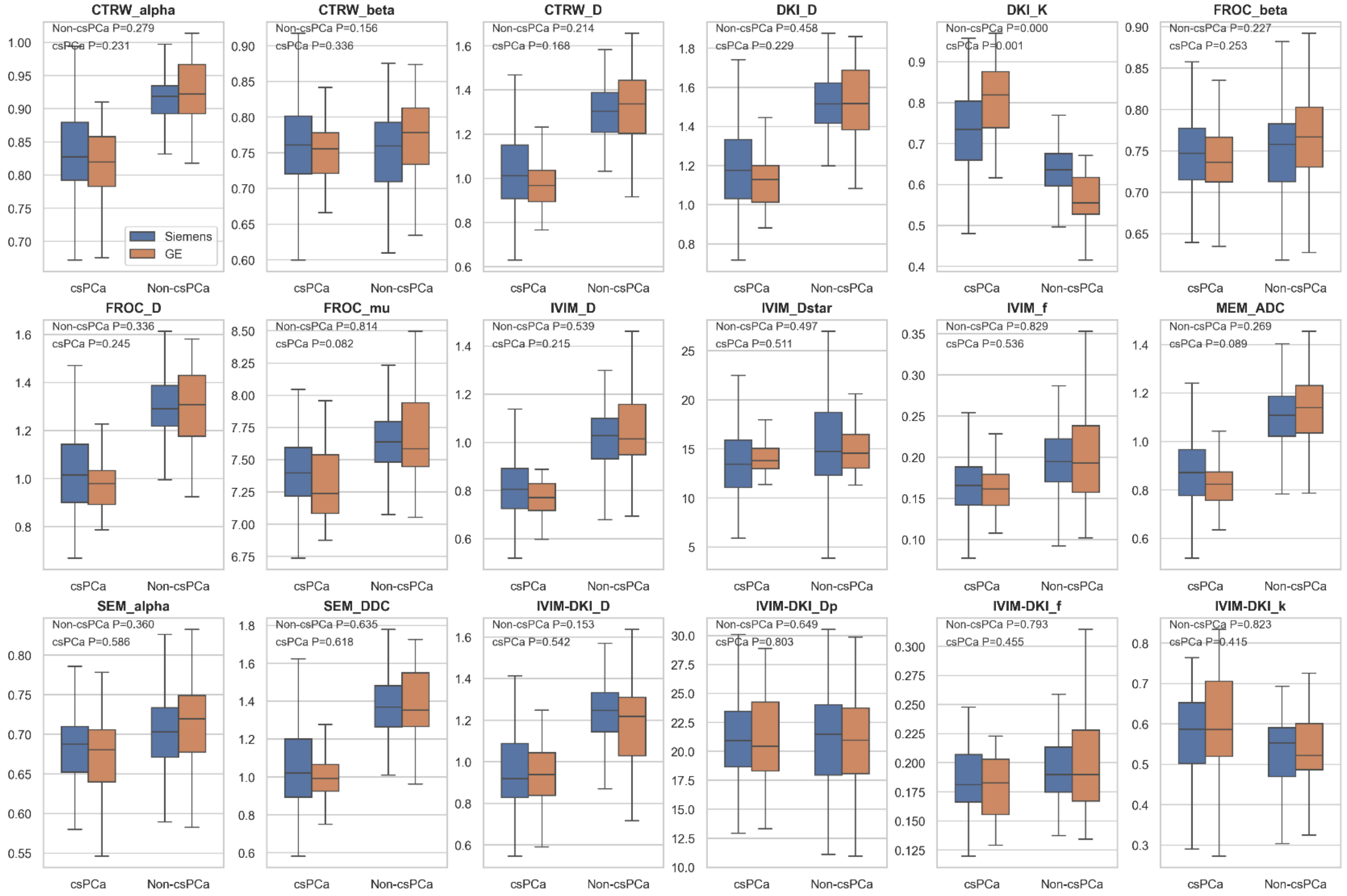

Insights Imaging (2026) He KW, Chen YS, Kang Z, et al.

Figure S7 Assessment of cross-vendor consistency for quantitative diffusion parameters following ComBat harmonization. Boxplots illustrate the distribution of 18 derived multi-b-value diffusion parameters compared between the primary (Siemens; blue) and independent cross-vendor (GE; orange) cohorts. Comparisons are stratified by pathological outcomes: non-clinically significant prostate cancer (Non-csPCa) and csPCa using Mann-Whitney U test. ComBat harmonization was performed prior to this analysis to mitigate scanner-specific technical variations.

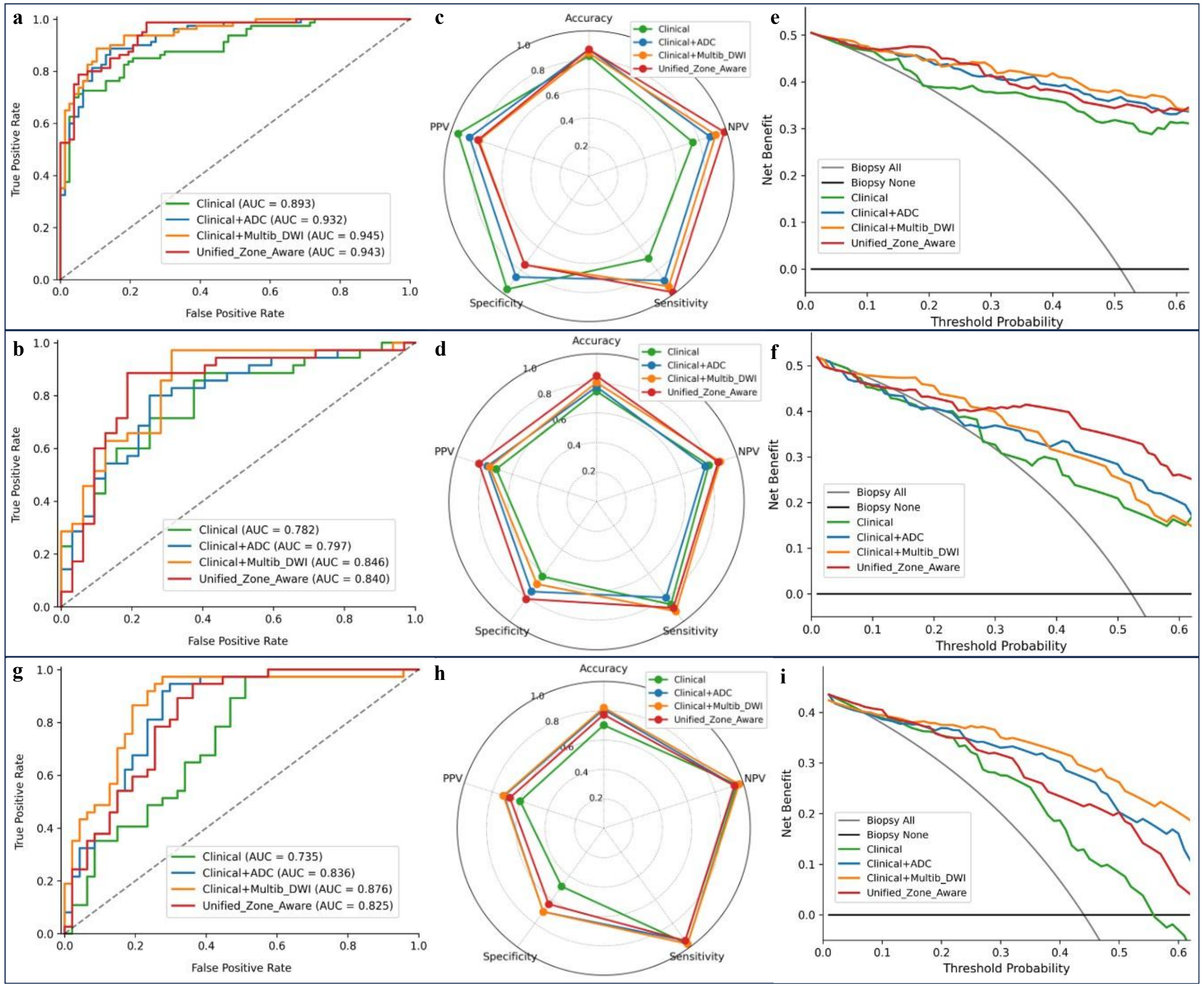
a
True Positive Rate
False Positive Rate
Clinical (AUC = 0.893)
Clinical+ADC (AUC = 0.932)
Clinical+Multib_DWI (AUC = 0.945)
Unified_Zone_Aware (AUC = 0.943)
c
Accuracy
NPV
Sensitivity
Specificity
PPV
Clinical
Clinical+ADC
Clinical+Multib_DWI
Unified_Zone_Aware
e
Net Benefit
Threshold Probability
Biopsy All
Biopsy None
Clinical
Clinical+ADC
Clinical+Multib_DWI
Unified_Zone_Aware
b
Clinical (AUC = 0.782)
Clinical+ADC (AUC = 0.797)
Clinical+Multib_DWI (AUC = 0.846)
Unified_Zone_Aware (AUC = 0.840)
d
f
g
Clinical (AUC = 0.735)
Clinical+ADC (AUC = 0.836)
Clinical+Multib_DWI (AUC = 0.876)
Unified_Zone_Aware (AUC = 0.825)
h
i

Figure S8. Diagnostic performance and clinical utility of the exploratory unified zone-aware model compared with the primary models. Receiver operating characteristic curves, radar plots, and decision curve analysis of the Clinical, Clinical+ADC, Clinical+Multib_DWI, and the exploratory Unified Zone-Aware models for distinguishing between clinically significant prostate cancer (csPCa) and non-csPCa in the training set (a, c, e), test set (b, d, f), and cross-vendor validation set (g, h, i).
